# Unstructured transcription factor interactions enable emergent specificity

**DOI:** 10.1126/science.aeb6487

**Published:** 2026-05-28

**Authors:** Abrar A. Abidi, Claudia Cattoglio, Natalie N. Tang, Vinson B. Fan, Gina M. Dailey, Amir D. Hay, Prasanthi Kunamaneni, Daniel E. Milkie, Xavier Darzacq, Eric Betzig, Robert Tjian, Thomas G. W. Graham

**Affiliations:** 1 Department of Molecular and Cell Biology, University of California, Berkeley, CA, USA; 2 Howard Hughes Medical Institute, University of California, Berkeley, CA, USA; 3 Thomas C. Jenkins Department of Biophysics, Johns Hopkins University, Baltimore, MD, USA; 4 Janelia Research Campus, Howard Hughes Medical Institute, Ashburn, VA, USA

## Abstract

How intrinsically disordered regions (IDRs) shape chromatin binding and nuclear organization of transcription factors (TFs) remains unclear. We used proximity-assisted photoactivation (PAPA), a single-molecule protein-protein interaction sensor, to investigate how IDRs might influence TF interactions with each other and with chromatin in live cells. We found that the Sp1 DNA binding domain (DBD) interacted poorly with chromatin and did not colocalize with Sp1. Weak interaction of the isolated IDR with full-length Sp1 was enhanced by fusion to various unrelated DBDs. Live imaging of *Drosophila* polytene chromosomes confirmed that an IDR could confer sharp locus specificity on an otherwise nonspecific DBD. These findings suggest that TF specificity emerges on chromatin when ensembles of diverse, unstructured interactions are scaffolded by transient DNA contacts.

## Introduction

For decades, transcription factor (TF) specificity has been attributed to structured DNA binding domains (DBDs) recognizing defined DNA sequence motifs on the genome (1–4). Yet this model conflicts with increasing experimental evidence (5, 6): Eukaryotic genomes contain vast numbers of potential binding sites, only a minority of which are occupied (7, 8); TFs with identical motif preferences occupy distinct genomic loci (9–11); and many occupied loci lack recognizable motifs altogether (12). These contradictions raise a central question: How do TFs achieve specificity on a genome where potential binding sites are both overabundant and underspecified?

Unlike bacterial TFs, eukaryotic TFs contain extensive intrinsically disordered regions (IDRs) (13), which do not fold into stable three-dimensional (3D) structures. IDRs participate in multivalent, low-affinity interactions whose native behavior in living cells has proved tremendously challenging to study. Recent work has suggested that IDRs influence TF genomic occupancy patterns (10, 14), but these conclusions rest on sequencing-based assays that infer binding indirectly from DNA cleavage signals. Previous imaging studies have relied on bulk fluorescence measurements that often involved ectopic overexpression, artificial multimerization, or synthetic binding arrays (15–17), which can substantially distort IDR behavior (18), leaving their role unclear.

In this study, we combined high-throughput oblique line-scan (OLS) microscopy (19) with proximity-assisted photoactivation (PAPA), a live-cell single-molecule imaging approach that can detect fleeting protein interactions (20–22). Studying the human TFs Sp1 and Klf1, we found that IDRs are required for chromatin binding and for TF self-interaction. The isolated Sp1 DBD showed markedly reduced chromatin binding and failed to colocalize with full-length Sp1. Replacing Sp1’s DBD with heterologous DBDs with distinct sequence preferences restored colocalization with wild-type (WT) Sp1, revealing an independence of locus specificity from sequence recognition. IDRs from functionally unrelated RNA binding proteins, which did not interact with Sp1 on their own, did so when fused to the Sp1 DBD, which demonstrates that DNA binding can facilitate otherwise unlikely unstructured interactions. Sp1 interacted with structurally diverse TFs and a transcriptional coactivator on chromatin—interactions that persisted even when Sp1’s intrinsic sequence preferences were lost. Extending these observations to living tissue, imaging of *Drosophila* salivary glands showed that fusion to an IDR caused a typically nonspecific DBD to acquire a sharply defined chromatin localization pattern.

Taken together, our findings show that DBDs alone do not determine where TFs bind on the genome. Instead, transient and underspecified DNA contacts scaffold ensembles of unstructured TF interactions. We propose a model of emergent specificity in which these dynamic interactions, although individually insufficient, collectively resolve into stable, locus-specific TF enrichments on chromatin.

## Results

### PAPA detects TF proximity in living cells

**Fig. 1.**
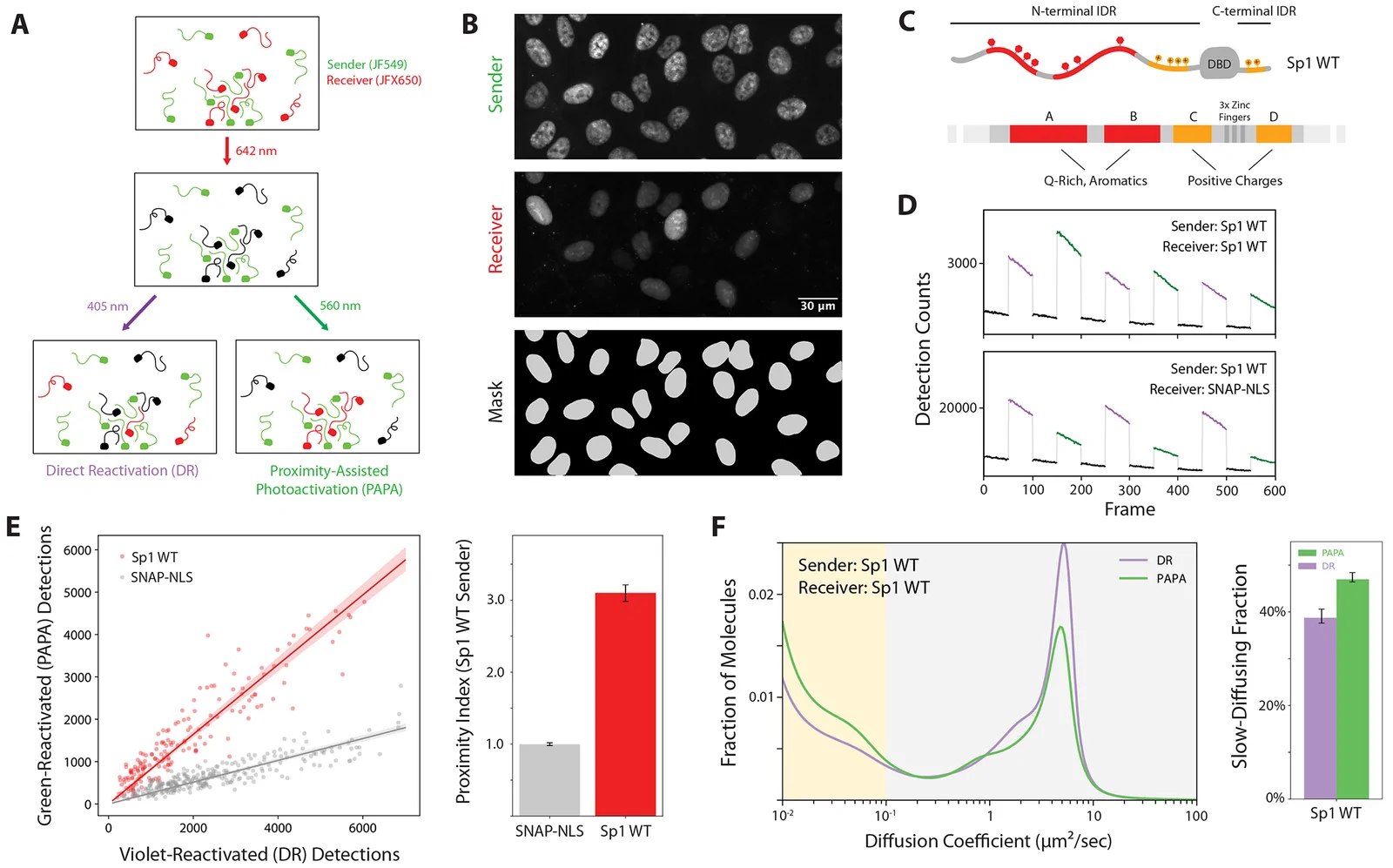
PAPA detects TF self-interaction in living cells. **(A)** Schematic of PAPA. Receiver-labeled proteins (red) are converted to a nonfluorescent dark state using 642-nm light. Subsequent excitation of nearby sender-labeled proteins (green) with 560-nm light selectively reactivates nearby receivers. As a control, 405-nm illumination induces DR of receivers independent of their proximity to the sender. **(B)** High-throughput imaging workflow on an OLS microscope. Representative images show Halo-conjugated sender fluorescence (JF549), SNAPf-conjugated receiver fluorescence (JFX650), and the nuclear segmentation masks used to define nuclei for downstream analysis. Doxycycline induction yields a subset of cells expressing the SNAPf-tagged protein. For single-molecule acquisition, receiver fluorophores are bleached down before imaging; the receiver channel is shown in this case before bleaching. **(C)** Domain diagram of Sp1. The N-terminal A and B regions are enriched in glutamine and aromatic residues (red hexagons), whereas the C and D regions flanking the DBD are enriched in basic residues (orange circles). Symbols schematize residue type and distribution, not exact counts or positions. **(D)** Detection counts aggregated over all cells per frame after alternating violet (DR) and green (PAPA) illumination for cells coexpressing Sp1 as both sender and receiver (top) or Sp1 with a noninteracting nuclear SNAPf control (bottom). **(E)** (Left) Scatterplot of DR versus PAPA detection counts per nucleus. (Right) Bar plot showing proximity index (PAPA/DR), normalized to noninteracting SNAPf-NLS control (see also fig. S1E). **(F)** (Left) Diffusion coefficient distributions of PAPA- and DR-reactivated molecules. (Right) Fraction of slow-diffusing (< 0.1 μm^2^/s) molecules reactivated by PAPA (green) and DR (violet). Localization error limits resolution of diffusion coefficients below ~0.01 μm^2^/s (19), and state array analysis assigns trajectories with D < 0.01 μm^2^/s into the lowest diffusion bin (21).

To use PAPA, Halo- and SNAPf-tagged proteins are labeled with JF549 “sender” and JFX650 “receiver” dyes, and receivers are placed in a photochemical dark state by excitation with red light (20). Excitation of senders with green light stochastically reactivates nearby receivers, revealing proximity between sender- and receiver-labeled molecules. Proximity-independent direct reactivation (DR) of receivers by violet light serves as an internal control (Fig. 1A). To increase throughput, we imaged cells on a custom-built OLS microscope that enables wide-field single-molecule imaging at high frame rates (Fig. 1B) (19). Although this method had previously been applied to stable, structured complexes, it was unclear whether it could resolve the weaker, transient contacts characteristic of IDRs. To test this, we examined the TF Sp1, which has a three–zinc finger DBD flanked by a long self-interacting N-terminal IDR (23–25) and a short C-terminal IDR (Fig. 1C).

We used Cas9 genome editing in human U2OS cells to insert HaloTag at the endogenous Sp1 locus (fig. S1, A and B) and a doxycycline-inducible SNAPf-Sp1 transgene at the AAVS1 safe harbor locus (fig. S1, A and C). Flow cytometry confirmed that transgene expression levels were comparable to or lower than that of endogenous Sp1, minimizing artifacts from overexpression (fig. S1D).

For each condition, we alternated imaging cycles with green and violet excitation and counted reactivated receiver localizations from single-molecule movies acquired at 10 ms per frame (Fig. 1, D and E). As a negative control, we also imaged Halo-Sp1 cells expressing noninteracting nuclear SNAPf (fig. S1A). We define a “proximity index” as the ratio of green- to violet-reactivated localizations for the protein pair of interest normalized to this negative control (Fig. 1E and fig. S1E). A proximity index of 1 indicates no detectable interaction above background, whereas values > 1 indicate that the proteins are within ~20 nm of each other more often than expected by chance. This metric thus distinguishes specific from incidental colocalization.

PAPA measured substantial Sp1 self-interaction in the nucleus (Fig. 1E), reproducible across many independent biological replicates (fig. S2, B and C). Single-molecule tracking (SMT) revealed that, compared with the overall population of molecules reactivated by DR, those reactivated by PAPA showed a greater proportion of slow-moving molecules in the diffusion range characteristic of H2B and other chromatin-bound proteins (fig. S4) (26–29). This suggests that Sp1 self-interaction enhances chromatin association.

We also examined Klf1, a TF not normally expressed in U2OS cells. Integrating a bicistronic transgene at the *AAVS1* locus, we coexpressed Halo-Klf1 with either SNAPf-Klf1 or a nuclear SNAPf control. Like Sp1, Klf1 showed considerable self-interaction (fig. S3). Taken together, these findings demonstrate that PAPA can be used on an OLS microscope to reproducibly measure transient TF interactions in living cells.

### The core Sp1 DBD is insufficient for chromatin binding and colocalization with full-length Sp1

**Fig. 2.**
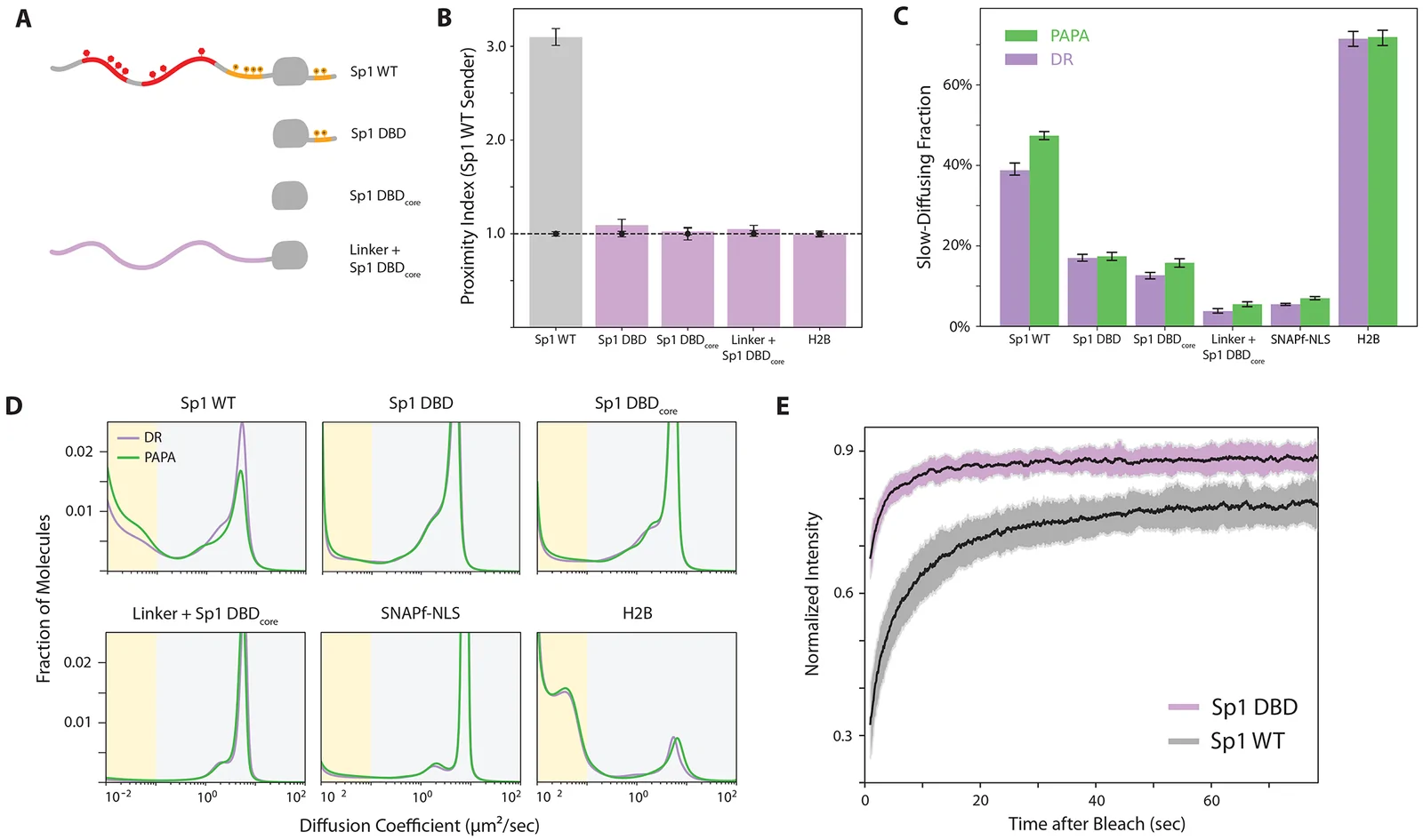
The isolated Sp1 DBD shows severe binding defects. **(A)** Domain diagrams of isolated DBD variants: N-terminal IDR deletion (Sp1 DBD), N- and C-terminal IDR deletion (Sp1 DBD_core_), and N-terminal linker-replacement (Linker + Sp1 DBD_core_). Red hexagons indicate aromatic residues, and orange circles indicate basic residues. Symbols schematize residue type and distribution, not exact counts or positions. **(B)** PAPA proximity index between sender-labeled full-length Sp1 and each receiver-labeled variant in (A), with histone H2B-SNAPf included as a noninteracting control. **(C)** Slow-diffusing (< 0.1 μm^2^/s) fraction of molecules reactivated by PAPA (green) and DR (violet) for each variant in (A), as well as SNAPf-NLS and H2B. **(D)** Diffusion coefficient distributions of each variant in (C) under DR and PAPA conditions. **(E)** FRAP recovery curves comparing full-length Sp1 (n = 25 cells) and Sp1 DBD (n = 26 cells), indicating reduced chromatin association.

Previous studies have shown that the structured Sp1 DBD binds GC-box motifs effectively *in vitro* (30, 31), and such motifs often occur in dense repeats throughout the human genome (32, 33), which suggests that the DBD alone should colocalize with WT Sp1 on chromatin. To test this, we expressed SNAPf-tagged deletion variants in the background of endogenous Halo-tagged Sp1 (Fig. 2A). Removing Sp1’s N-terminal IDR severely reduced chromatin binding (to 17% bound from 39% in WT) (Fig. 2, C and D, and fig. S4) and abolished colocalization with WT Sp1 (Fig. 2B). The core Sp1 DBD, with both N- and C-terminal IDRs removed, also failed to colocalize with WT Sp1 and showed even less chromatin binding (13% bound).

We asked whether either steric hindrance of DNA binding from SNAPf or loss of a flexible N-terminal extension needed to position the receiver fluorophore within PAPA’s detection range could explain why we could not measure colocalization. To rule these out, we inserted a flexible hydrophilic linker, nearly matching Sp1’s N-terminal IDR in length, between the DBD and SNAPf (Fig. 2A). This construct, which far better approximates WT Sp1 in both molecular weight and end-to-end polypeptide length, also failed to colocalize with full-length Sp1 (Fig. 2B) and showed a complete loss of chromatin binding (4%) (fig. S4), comparable to freely diffusing SNAPf (6%) (fig. S2). This total binding defect perhaps reflects the entropic cost imposed by an inert flexible polypeptide that lacks the stabilizing interactions provided by the WT IDR.

Next, we considered whether our baseline comparison—normalizing the proximity index to freely diffusing SNAPf—might underestimate proximity between Sp1 DBD and WT Sp1 on chromatin. As an independent negative control, we tested histone H2B, a core component of chromatin with no known interaction with Sp1. This produced an indistinguishable PAPA signal from freely diffusing SNAPf and the IDR-less Sp1 variants (Fig. 2B), which confirms that our baseline estimate is reliable.

Finally, we assessed chromatin binding using an independent method, fluorescence recovery after photobleaching (FRAP). This likewise showed acutely reduced binding by the DBD alone, as indicated by the markedly shallower bleach depth and faster recovery compared with WT Sp1 (Fig. 2E). Together, these results demonstrate that Sp1’s DBD is insufficient for either stable chromatin binding or colocalization with full-length Sp1 in live cells.

### Basic residues flanking the DBD stabilize chromatin binding

**Fig. 3.**
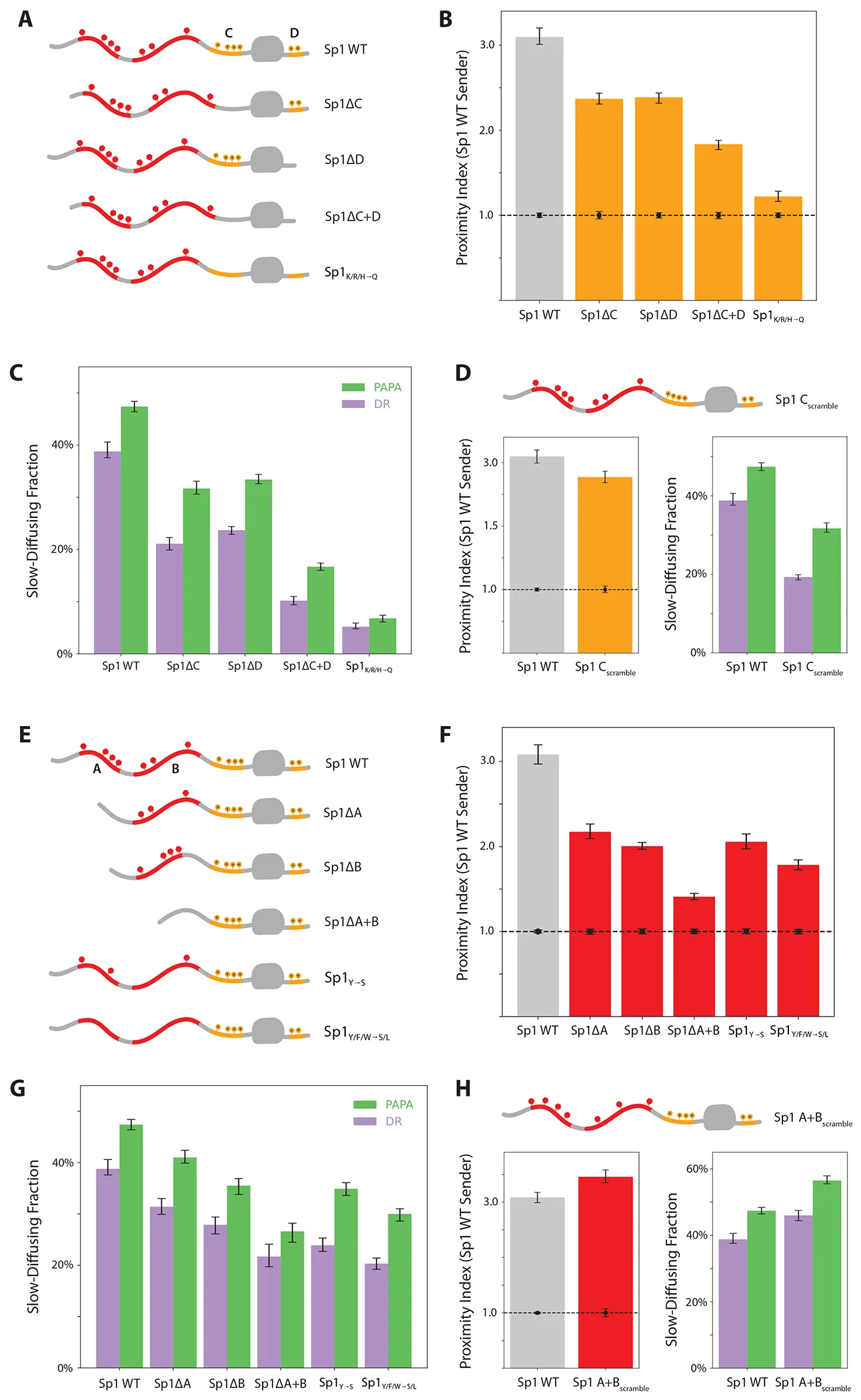
Basic residues flanking the Sp1 DBD stabilize chromatin binding, and aromatic residues mediate unstructured self-interaction. **(A)** Domain diagrams of C and D region deletion variants (Sp1ΔC, Sp1ΔD, Sp1ΔC+D) and a charge-neutralized IDR variant (Sp1_K/R/H→Q_). Red hexagons indicate aromatic residues, and orange circles indicate basic residues. Symbols schematize residue type and distribution, not exact counts or positions. **(B)** PAPA signal between full-length Sp1 and each variant in (A). **(C)** Slow-diffusing (< 0.1 μm^2^/s) fraction of molecules reactivated by PAPA (green) and DR (violet). **(D)** (Left) Effect of the C_scramble_ variant on PAPA with full-length Sp1. (Right) Corresponding slow-diffusing fractions. **(E)** Domain diagrams of A and B region deletion variants (Sp1ΔA, Sp1ΔB, Sp1ΔA+B) and aromatic-residue substitution IDR variants (Sp1_Y→S_, Sp1_Y/F/W→S/L_). **(F)** PAPA signal between full-length Sp1 and each variant in (E). **(G)** Slow-diffusing (< 0.1 μm^2^/s) fraction of molecules reactivated by PAPA (green) and DR (violet). **(H)** (Left) Effect of the A+B_scramble_ variant on PAPA signal with full-length Sp1. (Right) Corresponding slow-diffusing fractions.

We next examined the biochemical features of Sp1’s IDRs to understand how they might contribute to chromatin binding and genomic localization. Sp1 contains two positively charged unstructured subregions flanking its DBD: the N-terminal C region and the C-terminal D region (Fig. 3A). Clusters of basic residues adjacent to the DBD are a common feature of eukaryotic TFs (34), but their role in the nucleus remains poorly understood. Removing either region C (Sp1ΔC) or region D (Sp1ΔD) reduced chromatin binding and interaction with WT Sp1 (Fig. 3, B and C, and fig. S5). The double-deletion mutant (Sp1ΔC+D) showed a further loss of chromatin binding and WT colocalization, indicating that interactions mediated by regions C and D contribute additively. For all three mutants, PAPA-reactivated molecules were enriched for chromatin binding (Fig. 3C).

To separate the effects of IDR length from positive charge, we generated an Sp1 variant in which all lysine, arginine, and histidine residues outside the DBD were replaced with glutamine (Sp1_K/R/H→Q_) (Fig. 3A). This charge-neutralized mutant showed a total loss of chromatin binding (5%) and retained only minimal interaction with WT Sp1 (Fig. 3, B and C, and fig. S5).

To test the importance of the primary sequence of DBD-adjacent basic regions, we scrambled region C (Sp1C_scramble_) while preserving its overall residue composition (Fig. 3D). This reordering increased the average linear distance of basic residues from the DBD and substantially reduced chromatin binding (19%) but caused only a minor interaction defect with WT Sp1 (fig. S5). Together, these results suggest that basic unstructured regions adjacent to a structured DBD stabilize chromatin binding, likely through weak electrostatic contacts with nucleic acids.

### Unstructured TF interactions rely on aromatic residues

The long N-terminal IDR of Sp1 contains two glutamine- and aromatic-rich subregions, A and B (Fig. 1C and Fig. 3A). Using PAPA, we analyzed deletion variants lacking the A region (Sp1ΔA), the B region (Sp1ΔB), or both regions (Sp1ΔA+B) (Fig. 3E). Like the C and D deletions, A and B deletions reduced both chromatin binding and interaction with WT Sp1 (Fig. 3, F and G, and fig. S6). Whereas the chromatin binding defects of A and B deletions were weaker than those of the C and D deletions, the interaction defects were stronger, revealing that these properties are not strictly correlated.

To assess the role of aromatic residues—and to control for protein length—we mutated either all tyrosines (Sp1_Y→S_) or all aromatic residues (Sp1_Y/F/W→S/L_) outside the DBD to nonaromatic residues (Fig. 3E). Both mutants showed markedly reduced chromatin binding and interaction with WT Sp1 (Fig. 3, F and G). The tyrosine-less variant showed a milder defect compared with the aromatic-less variant, which indicates that homotypic interactions scale with total aromatic content.

To test whether the primary sequence of these regions—not just aromatic content—influences homotypic interactions, we scrambled regions A and B (Sp1A+B_scramble_) while preserving overall residue composition (Fig. 3H). This dispersed aromatic residues and reduced their clustering along the length of the polypeptide—an alteration predicted by simulation and *in vitro* studies to weaken self-interaction (35). Instead, the scrambled variant interacted more strongly with WT Sp1 and showed greater overall chromatin binding (fig. S6), which indicates not only that Sp1’s IDR tolerates sequence reordering but that even a simple reordering can enhance interactions.

The effect of residue reordering on interaction with WT Sp1 prompted us to explore whether naturally occurring sequence variation would show similar effects. Sp1 is highly conserved between humans and mice (96% sequence identity), but Klf1 is less so (71%), with most sequence differences in its N-terminal IDR. Although the total number of aromatic residues in this IDR is conserved between the two species, aromatic identity and positions differ slightly. Mouse Klf1 interacted more strongly with human Klf1 than human Klf1 did with itself, indicating that even modest evolutionary divergence in IDR composition can alter these interactions (fig. S3). Together, these results demonstrate that unstructured TF interactions in the nucleus are shaped by both the number and positional arrangement of aromatic residues within IDRs.

### Weak DNA binding stabilizes unstructured TF interactions

**Fig. 4.**
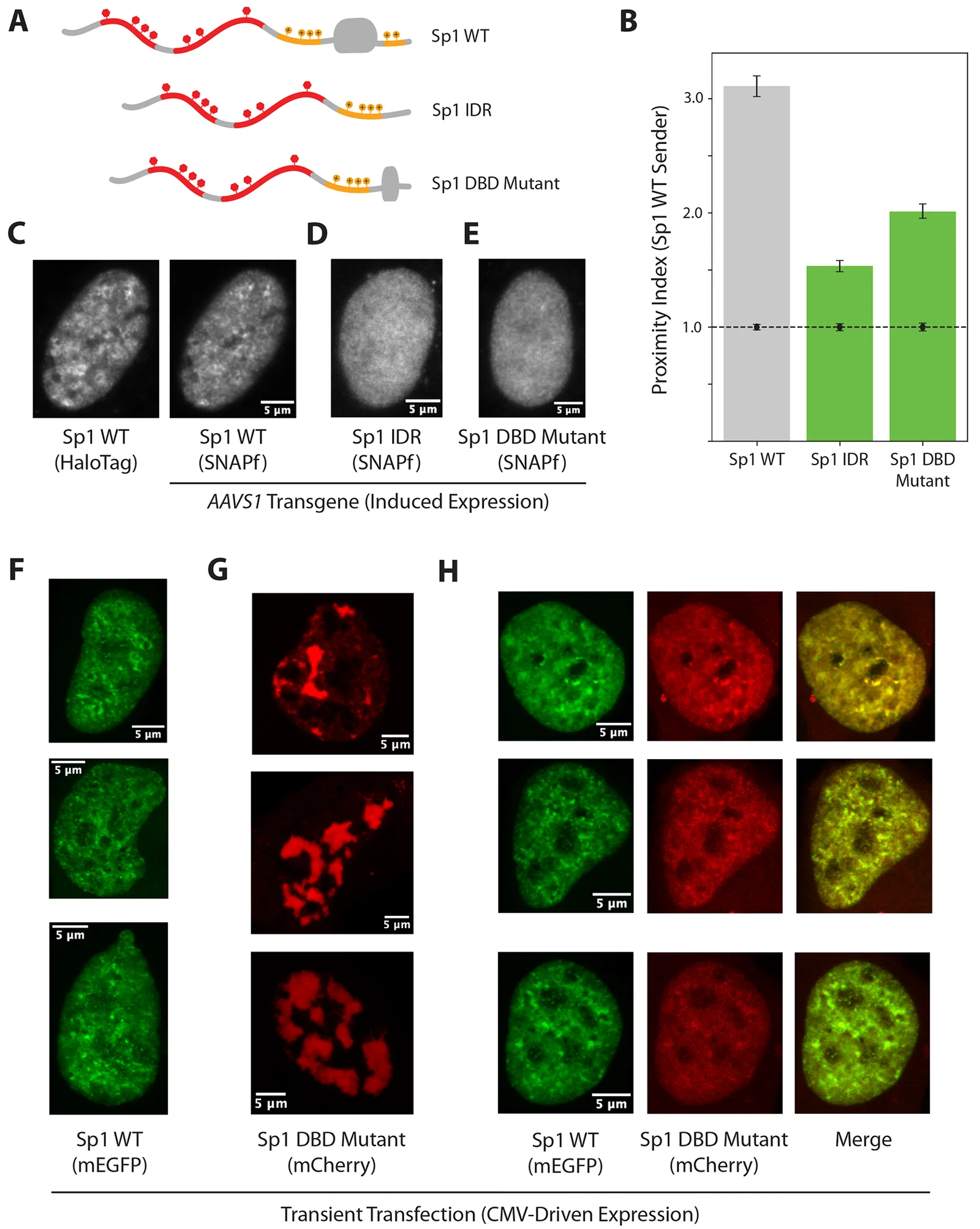
Sp1 IDR interactions persist in the absence of stable DNA binding. **(A)** Domain diagrams of the isolated N-terminal IDR (Sp1 IDR) and a single–zinc finger mutant (Sp1 DBD mutant). **(B)** PAPA proximity index between full-length Sp1 and variants. **(C to E)** Still images showing induced *AAVS1*-integrated SNAPf-tagged variants: Sp1 WT (C), Sp1 IDR (D), and Sp1 DBD mutant (E). The endogenous Halo-tagged Sp1 WT channel is also shown in (C). **(F to H)** Transient transfection of U2OS cells using CMV-driven constructs. (F) Monomeric enhanced green fluorescent protein (mEGFP)–labeled Sp1 WT. (G) mCherry-labeled Sp1 DBD mutant. (H) Cotransfection of Sp1 WT and Sp1 DBD mutant, including both channels merged.

Given the essential role of Sp1’s N-terminal IDR in both Sp1 self-interaction and chromatin binding, we next asked whether it retains these properties on its own. The isolated IDR retained a weak interaction with WT Sp1 (Fig. 4, A and B, and fig. S4). Similarly, the Klf1 IDR retained a weak interaction with full-length Klf1 (fig. S3).

We next examined a disease-associated Sp1 variant harboring a heterozygous frameshift that leaves the N-terminal IDR intact but truncates the DBD to a single zinc finger (Sp1 DBD mutant; Fig. 4A) (36). Because a lone zinc finger is expected to form only weak, nonspecific DNA contacts, we anticipated that this variant would behave similarly to the isolated IDR. Instead, it showed significantly greater interaction with WT Sp1 (Fig. 4B and fig. S4), suggesting that even weak, nonspecific DNA binding can stabilize unstructured TF interactions.

We wondered whether this finding extends to other disease-linked TF mutations. We examined two clinically relevant Klf1 variants: E325K, which causes dominant congenital dyserythropoietic anemia type IV (37), and K288X, which causes hereditary persistence of fetal hemoglobin (HPFH) (38). In E325K, a glutamate in the second zinc finger is replaced by lysine; this substitution is thought to alter DNA binding specificity, leading the mutant protein to bind an alternative motif and drive aberrant expression of noncanonical targets. In K288X, a truncation upstream of the DBD abolishes structured DNA binding, and the mutant protein is generally considered nonfunctional, with the disease attributed to Klf1 haploinsufficiency. We found that both mutants retained substantial interaction with WT Klf1 (fig. S3). This suggests that modification, or even complete loss, of sequence-specific DNA binding does not preclude mutant TFs from interacting with WT counterparts or potentially other partners through unstructured interactions.

### DNA binding and unstructured partner interactions offset phase separation

Given the extensive literature linking IDRs to liquid-liquid phase separation (LLPS) (15, 39, 40), it was notable that neither the isolated Sp1 IDR nor the Sp1 DBD mutant formed discernible nuclear puncta when expressed at or below the physiological expression level of endogenous Sp1 (Fig. 4, C to E). By contrast, the mutant formed large nuclear puncta when strongly overexpressed from a cytomegalovirus (CMV) promoter (Fig. 4G). WT Sp1 expressed from the same CMV promoter maintained a well-distributed, less heterogeneous nuclear pattern (Fig. 4F), resembling the pattern consistently observed for endogenous Sp1 (Fig. 4C).

When WT and mutant Sp1 were coexpressed from a CMV promoter, the mutant puncta disappeared, and the protein colocalized with WT Sp1, adopting a more evenly distributed nuclear pattern (Fig. 4H). These findings suggest that formation of large nuclear puncta by the mutant reflects self-interaction at high concentrations in the absence of stable DNA binding, whereas mutant interactions with the coexpressed DNA binding–competent full-length protein forestall unrestrained condensate formation. Although recent work has shown that DNA binding can directly counteract protein condensation (41), our results demonstrate that the same effect can arise indirectly through unstructured interactions.

### IDR-dependent TF colocalization on chromatin does not strictly depend on DBD sequence specificity

**Fig. 5.**
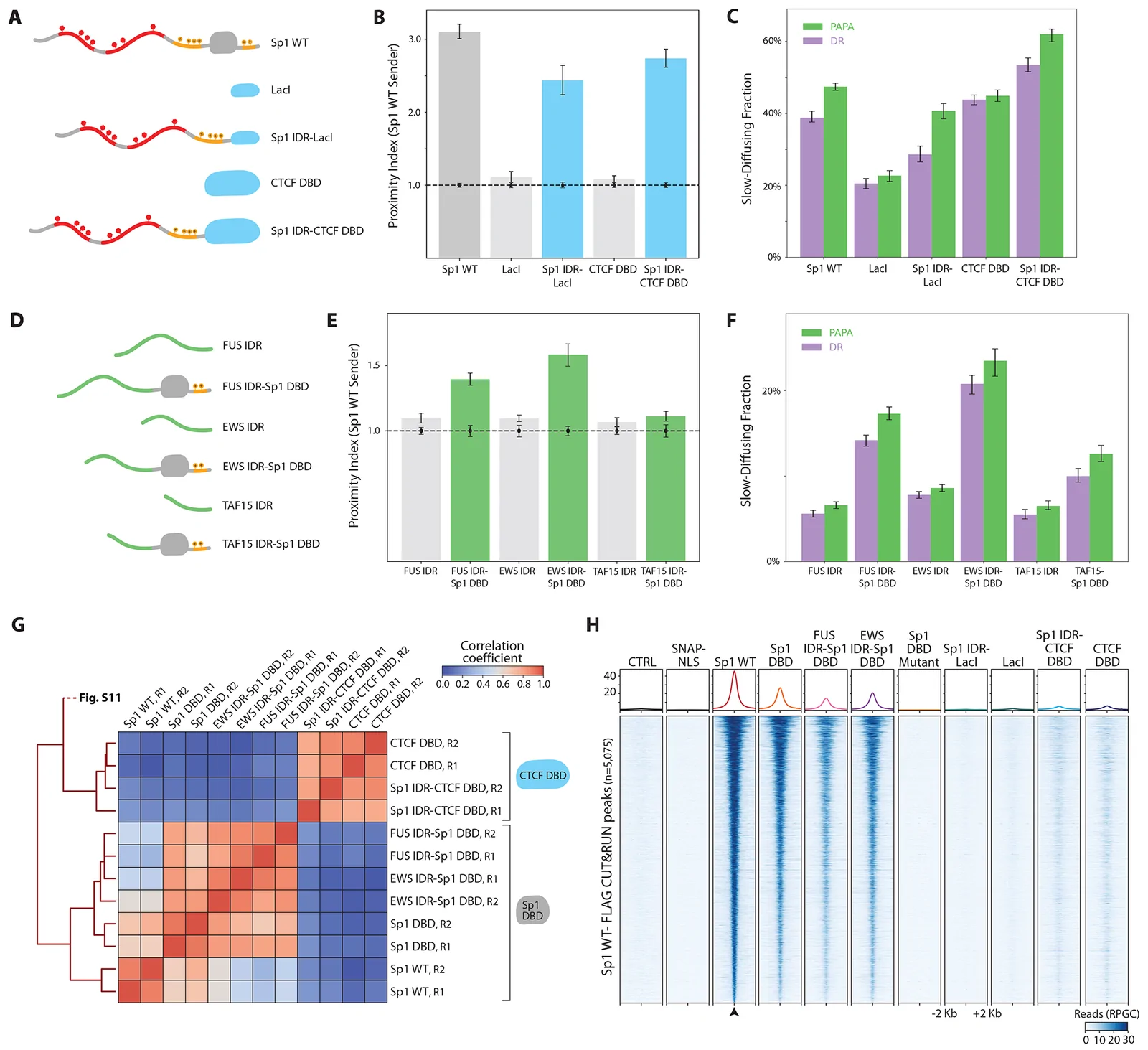
Domain swaps show that Sp1 genomic localization is largely independent of DBD sequence preferences. **(A)** Domain diagrams of chimeric variants in which the Sp1 DBD is replaced with LacI or the CTCF DBD, alongside LacI and CTCF DBD alone. Red hexagons indicate aromatic residues, and orange circles indicate basic residues. Symbols schematize residue type and distribution, not exact counts or positions. **(B)** PAPA signal between full-length Sp1 and each variant in (A). **(C)** Slow-diffusing (< 0.1 μm^2^/s) fraction of molecules reactivated by PAPA (green) and DR (violet) for each variant in (A). **(D)** Domain diagrams of chimeric constructs in which IDRs from FUS, EWS, or TAF15 replace the N-terminal IDR of Sp1, alongside the isolated heterologous IDRs. **(E)** PAPA signal between full-length Sp1 and each variant in (D). **(F)** Slow-diffusing fraction of PAPA- or DR-reactivated molecules for each variant in (D). **(G)** Heatmap of pairwise Spearman correlation coefficients of CUT&RUN genome coverage files computed on all reproducible FLAG CUT&RUN peaks (irreproducible discovery rate ≤ 0.1) for two independent biological replicates. R1, replicate 1; R2, replicate 2. See fig. S11 for the full all-versus-all correlation heatmap (including controls and LacI constructs). **(H)** Heatmap of CUT&RUN and ChIP-Seq read counts (reads per genomic content) ±2 kb around the center of 5075 reproducible FLAG peaks identified by CUT&RUN in the inducible SNAPf–Sp1 WT–FLAG line (irreproducible discovery rate ≤ 0.1). Plotted are CUT&RUN and ChIP-seq signals of the specified antibodies in the parental U2OS V5-Halo-Sp1 endogenous knock-in line (CTRL) and the inducible SNAPf–Sp1 WT–FLAG line derived from it. An arrowhead points to the sample used to sort the heatmap (highest to lowest peak intensity). RPGC, reads per genomic content.

Given that even a single zinc finger from the Sp1 DBD stabilized unstructured interactions (Fig. 4B), we wondered whether this effect extends to other DBDs. To test this, we replaced Sp1’s DBD with two structurally and functionally distinct alternatives: the DBD of human CTCF, which is highly sequence specific, and the *Escherichia coli* lac repressor (LacI), which has no cognate sequences in human cells and is thus thought to bind DNA nonspecifically (Fig. 5A). Both chimeras showed greater colocalization with WT Sp1 than the CTCF DBD alone, LacI alone, or Sp1’s N-terminal IDR alone (Fig. 4B, Fig. 5B, and fig. S7). Additionally, both chimeric constructs increased overall chromatin binding compared with the DBDs in isolation (Fig. 5C and fig. S7). PAPA-reactivated molecules were enriched for chromatin binding, which indicates that both chimeras interact with full-length Sp1 on chromatin. Together, these results suggest that structured DNA binding can stabilize unstructured interactions along chromatin—an effect that is influenced by but not strictly dependent on DBD sequence preferences.

To test whether chromatin binding can enhance IDR interactions more generally, we revisited a previous study that tethered the Sp1 IDR to a large synthetic gene array and found no colocalization with coexpressed, freely diffusing IDRs from the RNA binding proteins EWS, FUS, or TAF15 (17). Consistent with those findings, we observed little to no interaction between these isolated IDRs and endogenous Sp1 in live cells (Fig. 5E and fig. S8). However, when we replaced Sp1’s N-terminal IDR with any of these three heterologous IDRs (Fig. 5D), the resulting chimeras showed stronger chromatin binding than the IDRs in isolation (Fig. 5F and fig. S8). Notably, the FUS and EWS chimeras—but not the TAF15 chimera—acquired measurable interactions with Sp1 (Fig. 5E and fig. S8). These results suggest that concentrating IDRs on chromatin through structured DNA binding can promote interactions that do not occur between freely diffusing IDRs. Nonetheless, the FUS and EWS chimeras interacted far less with WT Sp1 than Sp1 did with itself, consistent with each IDR having a distinct continuum of interaction preferences.

### Genomic analyses diverge from live-cell measurements

To evaluate whether a standard sequencing-based assay captures the trends in TF localization that we observed in our live-cell single-molecule measurements, we performed cleavage under targets and release using nuclease (CUT&RUN). We first validated CUT&RUN by confirming strong concordance between endogenous and inducible Sp1 (fig. S10), high reproducibility across replicates (Fig. 5G and fig. S11), and agreement with a chromatin immunoprecipitation sequencing (ChIP-seq) dataset that we generated in parallel (fig. S10). We then profiled a panel of Sp1 variants.

Both full-length Sp1 and the isolated Sp1 DBD produced broadly overlapping CUT&RUN peaks (Fig. 5, G and H). The few peaks specific to either protein imply that the N-terminal IDR modestly influences—but does not fundamentally alter—Sp1’s genomic localization pattern. Consistent with this, replacing Sp1’s N-terminal IDR with that of EWS or FUS produced binding profiles closely resembling that of Sp1’s DBD alone (Fig. 5H), albeit showing slightly less overlap with full-length Sp1 (Fig. 5G). CUT&RUN therefore suggests that genomic localization is dominated by DBD sequence preferences. By contrast, PAPA showed no detectable colocalization between the Sp1 DBD and WT Sp1, despite the near-complete overlap reported by CUT&RUN.

To further examine this discrepancy, we profiled the Sp1 IDR–CTCF DBD chimera (fig. S13, A and B). Its CUT&RUN peaks mirrored those of the isolated CTCF DBD (Fig. 5G) and overlapped broadly with endogenous CTCF-bound regions (fig. S13A). A subset of these peaks overlapped with Sp1 peaks, and Sp1’s overlap with the Sp1 IDR–CTCF DBD chimera was indistinguishable from its overlap with the CTCF DBD alone. This again contrasts with PAPA results, which showed that only the chimera—not the CTCF DBD—measurably colocalized with WT Sp1 in live cells (Fig. 5B). One possible explanation for this divergence is that the CTCF chimera redistributed the genomic localization of endogenous Sp1 through unstructured interactions. To test this, we profiled endogenous Sp1 in cells coexpressing the CTCF chimera (fig. S14, A to C). However, CUT&RUN detected no substantial redistribution of endogenous Sp1’s binding peaks in the presence of the chimera.

PAPA also showed that an Sp1 IDR–LacI chimera colocalized with Sp1 at levels comparable to the Sp1 IDR–CTCF DBD chimera. By contrast, Sp1 IDR–LacI produced negligible CUT&RUN peaks and no enrichment at Sp1 sites, consistent with LacI alone (Fig. 5G and figs. S11 and S12). Likewise, the disease-associated Sp1 mutant retaining a single zinc finger produced no detectable CUT&RUN peaks (fig. S12), despite clear colocalization with Sp1 observed with PAPA and confocal imaging (Fig. 4, B and H).

Together, these results suggest that CUT&RUN overemphasizes the contribution of the DBD to chromatin binding while failing to capture binding and colocalization patterns evident in live cells. Whereas PAPA detects interactions directly in intact nuclei, CUT&RUN infers binding from DNA cleavage frequencies in dead and permeabilized cell populations. Consistent with this distinction, we found that the permeabilization step in CUT&RUN led to a substantial loss of Sp1 from the nucleus into the supernatant, an effect not observed for the much more stably chromatin-bound histone H2B (fig. S15). This raises the possibility that TF localization patterns that are robustly observed in living cells may be invisible to standard genomic assays.

### Live-tissue polytene chromosome imaging confirms IDR-dependent genomic localization

Although PAPA can reveal whether proteins colocalize on chromatin in live cells, it does not indicate where on the genome this occurs. To address this limitation, we imaged *Drosophila melanogaster* salivary gland cells, in which chromosomes undergo multiple rounds of endoreplication without mitosis, generating ~2000 aligned sister chromatids known as polytene chromosomes. Proteins enriched at specific loci occupy discrete bands within these ordered arrays, providing a visible readout of locus-specific binding in a living tissue.

**Fig. 6.**
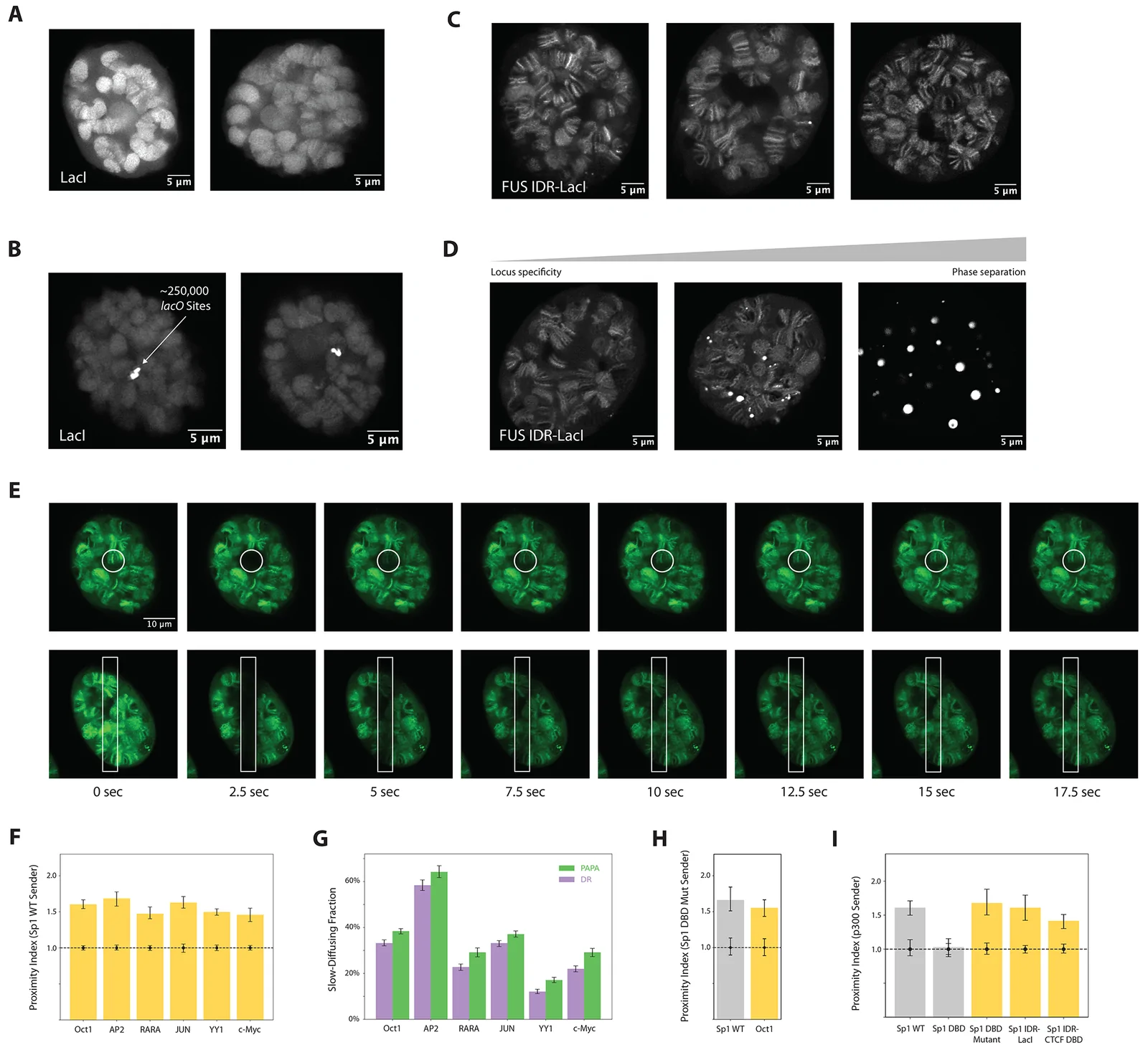
Heterotypic unstructured interactions influence TF localization. **(A**) Localization of LacI on polytene chromosomes in live *Drosophila* salivary gland cells. (A) to (C) show representative nuclei from two or three independent cells. **(B)** LacI enrichment at a lacO array (~250,000 sites per locus). **(C)** Localization of FUS IDR–LacI chimera on polytene chromosomes. **(D)** High-expressing cells show a progressive shift of FUS IDR–LacI from bands to putative phase-separated droplets. **(E)** FRAP recovery from genomic regions exhibiting banded localization by FUS IDR–LacI chimera. **(F)** PAPA proximity index between endogenous Sp1 and SNAPf-tagged heterotypic TFs. **(G)** Slow-diffusing (< 0.1 μm^2^/s) fraction of molecules reactivated by PAPA (green) and DR (violet) for the same factors as in (F). **(H)** PAPA proximity index between Halo-Sp1 DBD mutant and either coexpressed SNAPf-Sp1 WT or coexpressed SNAPf-Oct1. **(I)** PAPA proximity index between endogenous Halo-p300 and SNAPf-fusions of Sp1 WT, Sp1 DBD, Sp1 DBD mutant, Sp1 IDR–LacI, or Sp1 IDR–CTCF DBD. Display lookup tables are rescaled for clarity between panels (A) through (E) (see also figs. S17 to S19).

We began by visualizing nonspecific binding. As expected, LacI alone showed indiscriminate chromatin binding in salivary gland cells, with no discernible bands (Fig. 6A). As a control, bright bands were observed in flies heterozygous for a 256× lacO array (~250,000 lacO sites when polytenized; Fig. 6B), which confirms that LacI can bind specific sequences when they are present.

Building on our earlier finding that diverse IDRs, when coupled to DBDs, engage TFs on chromatin, we fused the human FUS IDR to LacI and expressed the chimera in *Drosophila* salivary glands. This fusion protein localized to discrete bands within decompacted, euchromatic regions of polytene chromosomes, revealing locus-specific enrichment despite the complete absence of lacO sites in the *Drosophila* genome (Fig. 6C and fig. S16). This banded localization pattern was observed whether FUS IDR–LacI was expressed at a lower or higher level compared with LacI (figs. S17 and S18), demonstrating that unstructured interactions can confer locus specificity on an otherwise nonspecific DBD.

At especially high transgene expression levels, FUS IDR–LacI formed large spherical puncta characteristic of LLPS droplets (Fig. 6D and fig. S19) (42). These large droplets did not localize to specific bands, and their formation coincided with the diminishment and ultimate disappearance of FUS IDR–LacI from bands. This mutual exclusivity suggests that phase separation disrupts rather than promotes IDR-dependent, locus-specific binding.

Consistent with our PAPA measurements of isolated IDRs, the FUS IDR alone was mostly diffuse within the nucleus but also showed some faint colocalization with FUS IDR–LacI bands on chromosomes (fig. S20). This suggests that fusion to a DBD stabilizes the otherwise weak locus-specific interactions of this IDR.

To measure the kinetics of FUS IDR–LacI within bands, we performed FRAP. Fluorescence recovered rapidly, restoring the original pattern of bands within seconds (Fig. 6E), which indicates that locus enrichment arises from transient interactions rather than stable chromatin binding. In the absence of direct DNA sequence recognition by LacI, heterotypic interactions of the FUS IDR with endogenous *Drosophila* proteins likely underlie the chimera’s localization pattern.

### Heterotypic interactions with diverse TFs and cofactors are DBD independent

To assess whether heterotypic unstructured interactions contribute more broadly to TF colocalization on chromatin, we returned to using PAPA in human cells to measure interactions between endogenous Sp1 and six structurally diverse TFs. All six interacted with Sp1 to varying extents (Fig. 6F), and the PAPA-reactivated fractions consistently showed a shift toward higher chromatin binding (Fig. 6G and fig. S9). We then coexpressed the Sp1 DBD mutant with either WT Sp1 or the confirmed heterotypic partner Oct1 in an Sp1 knockout background and found that both interactions persisted (Fig. 6H) despite the mutant’s loss of sequence-specific DNA binding. These results suggest that unstructured interactions among various TFs can coordinate their binding to chromatin.

Because Sp1’s N-terminal IDR was originally characterized as a transactivation domain, we investigated whether interactions of WT Sp1 with coactivators are detectable in live cells by PAPA and whether these interactions are preserved when its native DNA sequence preferences are lost. PAPA with endogenous Halo-tagged proteins revealed selective interaction of Sp1 with p300 but not with BRD4 (Fig. 6I and fig. S21). As expected, the Sp1-p300 interaction was abolished upon deletion of the N-terminal Sp1 IDR. Notably, three Sp1 variants lacking native sequence-specific DNA binding—a DBD mutant, a LacI DBD swap, and a CTCF DBD swap—retained p300 interactions indistinguishable from WT Sp1, whereas none interacted detectably with BRD4. Thus, a single TF IDR can sustain specific interactions with other TFs and selective coactivator engagement on chromatin independently of its DBD.

## Discussion

We found that unstructured regions of TFs substantially determine binding to other TFs and chromatin in living cells. Although DBDs and IDRs are notably underspecified individually, our observations suggest that they collectively produce precise patterns of localization through ensembles of dynamic, interdependent contacts—a model that we call emergent specificity. Such interactions must be weak because strong, self-sufficient contacts would collapse these ensembles into fixed geometric arrangements. We propose that, during eukaryotic evolution, quantitative reductions in stable stereospecific interactions permitted the elaboration of weaker, multivalent contacts across flexible surfaces, a shift that may have given rise to a qualitatively distinct mode of specificity. This mode differs from classic models of TF cooperativity defined by stable interactions between folded domains—models first articulated in studies of bacterial gene regulation and reinforced by decades of *in vitro* biochemical work. By contrast, the TF localization patterns that we observed are irreducible to the intrinsic properties of individual domains and emerge only within an intact nuclear environment, which underscores the need for continued advances in live-cell measurements.

Emergent specificity may explain paradoxes such as the functional nonequivalence of TFs with near-identical DBDs or occupancy at sites lacking recognizable motifs. It could also account for why IDRs expanded and diversified during eukaryotic evolution, whereas DBDs apparently became less specific and deterministic. Although DBD conservation is commonly invoked to argue that evolutionary changes in gene regulation are driven by cis-regulatory mutations and rearrangements (43), the outsize influence of unstructured interactions on locus specificity complicates that interpretation. From this perspective, the degeneracy of eukaryotic DBDs, relative to the high sequence specificity of most bacterial TFs, might have relaxed selective constraints on cis-regulatory sequences, allowing determinants of specificity to evolve within unstructured regions. Sequence variation in IDRs could therefore represent a potent and underappreciated substrate for adaptive change.

Our measurements show that basic unstructured regions flanking DBDs—a widespread feature of eukaryotic TFs (34)—can increase general affinity for chromatin, likely through electrostatic interactions with the DNA phosphate backbone. Mutations that alter basic residue content, including variants linked to disease (44–46), could thereby modulate TF localization by shifting the energetic balance of structured and unstructured protein and nucleic acid interactions. Scrambling part of Sp1’s N-terminal IDR increased chromatin association and interaction with WT Sp1, and substitution with unrelated IDRs partially restored colocalization, which argues against a proposed model (47) that IDRs directly recognize specific DNA sequences.

Our findings may also shed light on the molecular pathogenesis of disease-associated TF mutants. Oncoproteins in which TF DBDs are fused to non-TF IDRs, such as EWS-FLI1 (48) and FUS-CHOP (49), may dysregulate a broader and less straightforwardly predictable set of genes by co-opting interactions with native TFs and transforming collective binding patterns, rather than simply aberrantly activating genes specified by the parental DBD. Dominant heterozygous DBD mutations in TFs, including Sp1, Klf1, PAX6, FOXP2, GATA3, and TCF4 (50–53), might operate similarly. We found that a disease-associated Sp1 DBD mutant, retaining only a single zinc finger and lacking sequence-specific DNA binding (36), nonetheless colocalized with WT Sp1, as did the isolated N-terminal IDR of Sp1. In previous work, homozygous deletion of the Sp1 DBD partially preserved a differentiation phenotype that was lost upon complete Sp1 deletion (54, 55), indicating that significant functionality remains despite a loss of sequence-specific DNA binding. Likewise, we found that Klf1 variants such as E325K and K288X, generally interpreted as altering motif specificity (56, 57) or causing haploinsufficiency (38), retained colocalization with WT Klf1. Together, these results suggest that unstructured interactions represent an underrecognized axis of pathology that warrants closer scrutiny.

PAPA and SMT do not resolve genomic coordinates or distinguish direct DNA binding from indirect chromatin association. Genomic assays, such as ChIP-seq or CUT&RUN, could provide this positional information. However, CUT&RUN reports DBD-dominated binding patterns that disagree with our live-cell chromatin colocalization measurements. CUT&RUN also failed to detect binding of an Sp1 IDR–LacI chimera or Sp1 DBD mutant, both of which colocalized with full-length Sp1 by imaging. Permeabilization during CUT&RUN released the majority of Sp1 molecules from the nucleus, whereas histone H2B was retained, indicating preferential loss of weak TF interactions. Chemical fixation used in ChIP-seq likewise systematically distorts TF interactions (58, 59). Enzymatic footprinting in permeabilized nuclei has reported minutes-long TF residence times at synthetic high-affinity arrays (60), in contrast to the seconds-scale residence times typically observed in live-cell imaging. These sequencing-based methods may therefore selectively capture long-lived structured interactions that survive lethal permeabilization or fixation treatments. Even if such stable interactions occur in intact nuclei, there is no *a priori* reason to assume that they are primarily responsible for transcriptional activation. Addressing the gap between genomic and live-cell measurements will better inform the appropriate interpretation of each class of assays.

Imaging of *Drosophila* salivary glands provides a distinctive way to visualize locus-specific TF binding in living tissue. Fusion of a nonspecific DBD to an IDR caused it to localize to discrete chromosomal bands. Similar observations have been reported for the RNA polymerase II C-terminal domain (61) and an endogenous *Drosophila* TF (62). The localization pattern that we observe likely arises from interactions with other nuclear proteins. Consistent with this, PAPA in human cells demonstrated that both WT Sp1 and a DNA binding–deficient mutant interact heterotypically with structurally diverse TFs, showing that these interactions are sustained even in the absence of DBD sequence specificity.

We found no evidence that TFs phase separate at physiological concentrations. The Sp1 DBD mutant formed puncta only when grossly overexpressed, and coexpression with WT Sp1 dissolved these puncta, causing the mutant to adopt the nuclear distribution of the full-length protein. On *Drosophila* polytene chromosomes, synthetic TF localization to bands was mutually exclusive with droplet formation. Together, these results argue that phase separation precludes locus-specific TF binding by disrupting subcritical unstructured interactions. The observed local chromatin enrichments may better resemble the “higher-order transient structures” described in recent studies of membrane signaling (63, 64).

Although our results link unstructured interactions to locus specificity and selective coactivator engagement, we do not reveal underlying genomic positions and we do not demonstrate that IDR-mediated TF localization activates gene expression. More broadly, interactions among the ~1600 other human TFs and their coactivators remain to be explored (65). Evolving tools such as proximity labeling (66) may accelerate this effort. A deeper understanding of unstructured interactions in live cells will be crucial to explaining how this higher-order mode of TF localization shapes tissue-specific gene expression patterns.

## Materials and methods

### Cell culture and cell line generation

U2OS cells were cultured at 37°C with 5% CO2 in high-glucose Dulbecco’s modified Eagle’s medium (DMEM) (Thermo Fisher no. 12800082) supplemented with 10% fetal bovine serum (FBS) and penicillin/streptomycin. Cells were routinely tested for mycoplasma contamination by polymerase chain reaction (PCR). To insert a HaloTag on endogenous Sp1, cells were cotransfected with a homology donor plasmid and a plasmid coexpressing Cas9 and a single guide RNA (sgRNA). Cells were stained with a Janelia Fluor Halo ligand, and individual Halo-positive cells were flow-sorted into 96-well plates. Homozygous knock-in clones were validated by PCR, Western blot, and sequencing. Among validated clones, D5 was selected and used for all subsequent experiments. For Sp1 knockout, cells were cotransfected with plasmids expressing Cas9 and sgRNAs targeting sequences flanking the Sp1 coding region. Venus-positive cells (reporting Cas9 plasmid delivery) were flow-sorted into 96-well plates, and deletion clones were validated by PCR and Western blot. Endogenous HaloTag-fusion cell lines of BRD4, CTCF, and p300 were described previously (21, 27, 67).

Doxycycline-inducible transgenes were inserted into the *AAVS1* safe harbor locus together with a linked puromycin resistance cassette. All SNAPf-tagged transgenes contained C-terminal 3xFLAG and 3xSV40 NLS sequences, and all Halo-tagged transgenes contained C-terminal V5 and 3xSV40 NLS sequences. Stable integrants were selected in medium supplemented with 1 μg/ml puromycin.

### OLS microscopy

We constructed an OLS microscope on a Nikon Ti2 E inverted motorized microscope stand equipped with an SR Plan Apo IR 60× NA 1.27 water immersion objective lens. Laser lines (405 nm, 488 nm, 560 nm, and 642 nm) were combined using dichroic mirrors, directed through an acousto-optic tunable filter (AOTF), and passed through a single-mode optical fiber using two achromatic FiberPorts (Thorlabs PAF2-A7A). A light sheet was generated using a Powell lens (Laserline Optics, LOCP-8.9R10-1.2) and swept over the sample using a 1D galvo mirror (Thorlabs GVS201). A field-programmable gate array (FPGA) controlled by custom LabVIEW software was used to modulate the AOTF and sweep the light sheet in synchrony with the exposure of a Hamamatsu Orca Fusion BT camera running in lightsheet mode. Samples were kept at 37°C with 5% CO2 within an OKO Labs stage incubation chamber.

### PAPA-SMT

Cells were plated in 35-mm MatTek glass-bottom dishes in phenol red-free DMEM and induced for 36 hours with 1 μg/ml doxycycline and simultaneously stained with 50 nM of JF549 Halo ligand and 50 nM of JFX650 SNAP ligand. Before imaging, cells were washed briefly in 1X phosphate-buffered saline (PBS), destained twice for 30 min with phenol red-free DMEM, and transferred to fresh phenol red-free DMEM (Gibco no. 31053036).

For OLS imaging, an automated script was used to acquire data across multiple 2304-pixel–by–1024-pixel fields of view per condition. Receiver fluorophores (JFX650) were converted to a nonfluorescent dark state by illuminating the sample with 642-nm light at full power (2.2 W at the laser head). Movies were recorded under continued 642-nm illumination at a frame rate of 10 ms per frame. After acquiring 50 baseline frames, the sample was illuminated with either 405-nm light at full power (100 mW at the laser head) or 560-nm light at 50% AOTF (1.1 W at the laser head) for 50 frames, and 50 more frames were imaged to detect reactivated fluorophores. Receiver dyes were then reconverted to the nonfluorescent dark state using a second pulse of 642-nm light for 50 frames, and this sequence was repeated for three cycles, alternating between 405 nm (DR) and 560 nm (PAPA).

Laser output through the objective was measured before the experiment using a slide-mounted power sensor (Thorlabs S170C). At settings compatible with the sensor’s detection range, measured powers were approximately 0.65 mW for 405 nm (full power), 22 mW for 560 nm (5% AOTF), and 30 mW for 642 nm (200-mW laser head power). These measurements served to validate the consistency of laser output across sessions. To verify measurement reproducibility, a positive control (Halo-Sp1 + SNAPf-Sp1) and negative control (Halo-Sp1 + SNAPf-NLS) were typically imaged at the beginning and end of each imaging session.

### PAPA-SMT data analysis

Molecules were tracked as previously described using quot (https://github.com/alecheckert/quot), a Python toolkit for high-accuracy single-molecule localization and trajectory analysis. Nuclei were segmented using the Cellpose algorithm (68), applied to 560-nm fluorescence images after Gaussian smoothing (σ = 10), with an estimated nuclear diameter of 80 pixels. Segmented regions smaller than 5000 pixels in area were excluded, and adjacent or touching nuclei were separated using a custom boundary-drawing routine. Nuclear masks were overlaid on 642-nm intensity images to apply single-cell intensity filters. Cells lacking above-background nuclear 642-nm signal were excluded to remove nonexpressing SNAPf-fusion cells. Trajectory data were intersected with nuclear masks to assign detections to individual cells (sample sizes for PAPA-SMT experiments listed in table S1).

To calculate proximity indices (fig. S1E), single-molecule detections within segmented nuclear masks were quantified before and after violet and green excitation pulses. In each imaging cycle, 50 baseline frames were acquired to measure spontaneous, illumination-independent reactivation of JFX650. After either a violet (DR) or green (PAPA) pulse, 50 additional frames were collected to quantify light-induced reactivation. For each pulse type and cycle, baseline detections were fit to a single-exponential decay model and extrapolated into the corresponding postpulse window to estimate spontaneous background reactivation. The projected background was subtracted from observed postpulse detections. Background-subtracted detections were summed across imaging cycles and across cells, and the total number of green-induced (PAPA) detections was divided by the total number of violet-induced (DR) detections to yield a green-to-violet (GV) ratio for each condition. Experimental GV ratios were normalized to the mean GV ratio of a noninteracting SNAPf-NLS negative control to compute the final proximity index. Statistical uncertainty was estimated by bootstrap resampling of individual cells with replacement (500 resamples per condition), recalculating the pooled GV ratio for each bootstrap sample. Error bars represent the 2.5th and 97.5th percentiles of the resulting bootstrap distribution. Uncertainty in the control condition is shown as an error bar centered at proximity index = 1.

To visualize the relationship between DR- and PAPA-reactivated molecules at the single-cell level, we plotted background-corrected green-induced (PAPA) detections against background-corrected violet-induced (DR) detections for each cell, summing detections across three imaging cycles. For each pulse type and cycle, baseline reactivation was estimated from the 50 frames preceding the pulse and subtracted from detections in the 50 frames after the pulse; negative values were truncated to zero. To account for experiment-to-experiment variation in overall reactivation efficiency, green detections were normalized by a control GV ratio computed from pooled control data using exponential background projection. A linear regression constrained through the origin was performed to quantify proportionality between green and violet signals. Ninety-five percent confidence intervals for the fitted slope were estimated by bootstrap resampling of individual cells (2000 resamples per condition).

Diffusion coefficient distributions were inferred using SASPT (https://github.com/alecheckert/saspt), a Bayesian state array framework for modeling heterogeneous mobility states from single-molecule trajectories. DR and PAPA trajectories were analyzed separately, using only the 50 postpulse frames within each cycle. The bound fraction (D < 0.1 μm^2^/s) was estimated for each bootstrap replicate, and error is reported as the 95% confidence interval across 96 bootstrap resamples.

### Live-cell imaging

WT U2OS cells were plated in 35-mm MatTek glass-bottom dishes in phenol red-free DMEM. Transfections were performed using FuGENE HD at a 4:1 reagent:DNA ratio. Three conditions were tested: (i) 2 μg pCMV-mEGFP-Sp1WT, (ii) 1 μg pCMV-mEGFP-Sp1WT + 1 μg pCMV-mCherry-Sp1Mut, and (iii) 2 μg pCMV-mCherry-Sp1Mut. Cells were imaged ~24 hours after transfection on an LSM 900 confocal microscope equipped with an Airyscan detector (Zeiss, Thornwood, NY). Imaging was carried out on a 37°C temperature-controlled stage with humidified air in a heated incubation chamber. High-resolution images were acquired with the Airyscan detector and processed using ZEN microscopy software (Zeiss). Images were postprocessed in Fiji by applying a consistent look-up table (LUT) range for each condition that preserved low-intensity signal.

### FRAP

Mammalian cell FRAP experiments were performed using a Zeiss LSM 900 confocal microscope with a 40× oil immersion objective. Sp1 knockout cells were induced for 36 hours with 1 μg/ml doxycycline to express either Halo-Sp1 WT or Halo-Sp1 DBD and labeled overnight with 50 nM Janelia Fluor 549 dye. Cells were imaged using a 561-nm laser with the pinhole set to its maximum aperture. For each measurement, the imaging region was set to include the entire nucleus, and imaging was conducted at scan speed 9 with unidirectional scanning and 8-bit depth. Image sequences were acquired at ~1.5 frames per second (average frame interval 0.64 s) for a total of 140 frames (90 s). Photobleaching was applied to a 2-μm-diameter circular region on frame 16 using the maximum 561-nm laser power, after a 15-frame prebleach baseline period used for normalization.

Salivary gland FRAP experiments were performed using a 488-nm laser. Image sequences were acquired at ~2 frames per second (average frame interval 0.5 s) with photobleaching applied on frame 6 using the maximum 488-nm and 561-nm laser powers.

### FRAP data analysis

FRAP movies were analyzed using a custom Python pipeline (https://github.com/vinsfan368/FRAPpy). A maximum intensity projection of each movie was used to generate a nuclear mask, which was smoothed and thresholded to segment the nucleus. The position of the bleach spot was extracted from CZI file metadata and corrected for lateral drift using 2D image cross-correlation. For each frame, the mean fluorescence intensity within the bleach spot was measured. Background signal was estimated as the median pixel intensity outside the nuclear mask. Recovery was calculated as the background-subtracted intensity ratio of the bleach spot to the full nucleus. This ratio was normalized to the average ratio over the prebleach frames (frames 1 to 15), such that full recovery corresponds to a normalized value of 1.

### Polytene chromosome imaging

*D. melanogaster* lines were maintained on standard Bloomington cornmeal medium at room temperature. P-element transformation was used to generate lines expressing mNeonGreen-LacI and mNeonGreen–FUS IDR–LacI under the control of a GAL4-inducible 5xUAS-hsp70 promoter. Embryo injections were performed by Rainbow Transgenic Flies, Inc. Transformants were identified using a white (+) marker and were mapped to chromosomes and balanced using standard genetic methods.

UAS transgene lines were crossed to the salivary gland-specific sgs3-GAL4 driver line (Bloomington Drosophila Stock Center no. 6870) or the inducible GeneSwitch-GAL4 GSG952 line (Bloomington Drosophila Stock Center no. 40984). For the reduced-expression experiment in fig. S18B, GeneSwitch-GAL4 was induced by addition of 500 μl of a 0.1 μg/ml solution of mifepristone (MedChemExpress HY-13683-50 mg) in water to the surface of the fly food in a 27-mm-diameter vial first- and second-instar larvae. Wandering third-instar larvae were dissected in Grace’s insect medium (Fisher Scientific no. 11-605-094) supplemented with an antibiotic-antimycotic mixture (Fisher Scientific no. 15-240-096). Dissected glands were transferred to a MatTek dish with a 14-mm central aperture (P35G-1.5-14-C). As described previously (69), a razor blade was used to clip the bottom legs off a Millicell Standing Cell Culture Insert (Millipore no. PI8P01250), which was then gently placed over the glands to hold them in place. In some cases, mineral oil was pipetted around the insert, and the central well was filled with Grace’s medium. Glands were imaged immediately after dissection on a Zeiss LSM 700, LSM 800, or LSM 900 microscope. The latter two instruments were equipped with Airyscan detectors, and Airyscan deconvolution was performed using the manufacturer’s default settings.

### Fluorescent SDS–polyacrylamide gel electrophoresis (SDS-PAGE)

Cell lines were induced with doxycycline and stained as described for PAPA-SMT experiments. Cells were then trypsinized, resuspended in phenol red-free DMEM, counted using a Countess 3 cell counter (Thermo Fisher), pelleted by centrifugation, and frozen at −80°C. Following a previously described protocol (21), pellets were thawed on ice, resuspended in dye-free SDS loading buffer [50 mM Tris-HCl, pH 6.8, 100 mM dithiothreitol (DTT), 2.5% beta-mercaptoethanol, 2% SDS, 10% glycerol], and clarified by passing 10 times through a 26-gauge needle. These lysates were heated to 95°C for 5 min, and a volume corresponding to 50,000 cells was loaded in each lane of a 10% Bis-Tris SDS-PAGE gel. JFX650 signal (SNAP ligand) was imaged in the “IR700” channel on a LI-COR Odyssey CLx Imager, and JF549 signal was imaged in the “Cy3” channel on a Bio-Rad Pharos FX Plus Molecular Imager using the “Low Sample Intensity” setting.

### Western blotting

To confirm the U2OS endogenous Sp1 HaloTag fusion or knockout, cells were lysed directly in SDS sample buffer by pelleting ~1 × 10^6^ cells from a 6-well plate, resuspending in 100 μl of SDS buffer, and heating at 95°C for 10 min. Lysates were resolved by SDS-PAGE and transferred to nitrocellulose membranes using wet transfer at a constant current of 90 mA overnight in transfer buffer (15 mM Tris-HCl, 20 mM glycine, 20% methanol, 0.0375% SDS). Membranes were briefly stained with Ponceau S to verify transfer, then blocked for 1 hour at room temperature in TBST containing 5% bovine serum albumin (BSA) with gentle agitation. Primary antibody incubation was carried out overnight at 4°C in TBST containing 5% BSA. To detect Sp1, membranes were incubated with anti-Sp1 mouse monoclonal antibody (Santa Cruz Biotechnology, sc-17824). After three washes in TBST, membranes were incubated for 1 hour at room temperature with a horseradish peroxidase (HRP)–conjugated anti-mouse secondary antibody (1:5000 dilution in 5% BSA in TBST). Blots were then washed three times in TBST with brief (~2-min) incubations under agitation before chemiluminescent detection.

For measurement of nuclear protein depletion resulting from permeabilization during CUT&RUN, U2OS endogenous V5-Halo-Sp1 knock-in cells were stained with 100 nM JFX650 HaloTag ligand for 1.5 hours at 37°C, washed twice briefly with growth medium, and destained in fresh medium for an additional 1 hour. Stained cells and an unstained control were then trypsinized and counted. For each condition, 8 × 10^5^ cells were used, with 4 × 10^5^ cells reserved as input and 4 × 10^5^ cells used to assess TF leakage. Cells were subjected to a mock CUT&RUN protocol and incubated overnight at 4°C with normal rabbit immunoglobulin G (IgG) in 100 μl digitonin wash buffer containing 0.02% digitonin. The next day, pAG-MNase was added directly to input samples, and supernatants from the remaining samples were collected and Concanavalin A beads were washed twice with 100 μl digitonin wash buffer before pAG-MNase addition. After 1 hour of MNase binding at 4°C, CaCl2 was added and samples were digested on ice for 30 min. Aliquots (60 μl) of digested input, supernatant, wash 1, wash 2, and MNase-digested material were mixed with an equal volume of SDS-PAGE loading buffer lacking bromophenol blue, heated at 95°C for 6 min, and resolved on 9% Bis-Tris polyacrylamide gels (25 μl per lane). JFX650 HaloTag ligand signal was visualized using a LI-COR Odyssey CLx Imager. For Western blot analysis, gels were transferred to nitrocellulose membranes (Amersham Protran, 0.45 μm) at 100 V for 1.5 hours, blocked for 1 hour at room temperature in TBST containing 10% milk, and incubated overnight at 4°C with primary antibodies diluted in TBST containing 5% milk. HRP-conjugated secondary antibodies were diluted 1:5000 in TBST containing 5% milk and incubated for 1 hour at room temperature before chemiluminescent detection. Band intensities were quantified using Fiji (Gel Analyzer plugin).

### Chromatin immunoprecipitation and ChIP-seq library preparation

ChIP was performed as described (58) with some modifications. Endogenously tagged knock-in clones C32 (FLAG-Halo-CTCF, ATCC CRL-3625) and V5-Halo-Sp1 were expanded to four 15-cm dishes and cross-linked 6 min at room temperature with 1% formaldehyde-containing FBS-free medium; cross-linking was stopped by adding PBS-glycine (0.125 M final). Cells were washed twice with ice-cold PBS, scraped, centrifuged for 10 min and pellets were flash-frozen. Cell pellets were thawed and resuspended in 2 ml of cell lysis buffer (5 mM PIPES, pH 8.0, 85 mM KCl, and 0.5% NP-40; 1 ml per 15-cm plate) with protease inhibitors and incubated for 10 min on ice. Lysates were centrifuged for 10 min at 4000 rpm and nuclear pellets resuspended in 6X volumes of sonication buffer (50 mM Tris-HCl, pH 8.1, 10 mM EDTA, 0.1% SDS) with protease inhibitors, incubated on ice for 10 min, and sonicated to obtain DNA fragments around 500 base pairs (bp) in length (Covaris S220 sonicator, 20% Duty factor, 200 cycles/burst, 150 peak incident power, 10 to 13 cycles 30 s on and 30 s off). Sonicated lysates were cleared by centrifugation, and chromatin (300 to 400 μg per antibody) was diluted in radioimmunoprecipitation assay (RIPA) buffer (10 mM Tris-HCl, pH 8.0, 1 mM EDTA, 0.5 mM EGTA, 1% Triton X-100, 0.1% SDS, 0.1% Na-deoxycholate, 140 mM NaCl) with protease inhibitors to a final concentration of 0.8 μg/μl, precleared with Protein G dynabeads (Fisher 10009D) for 2 hours at 4°C and immunoprecipitated overnight with 3 to 4 μg of specific antibodies. About 4% of the precleared chromatin was saved as input. Immunoprecipitated DNA was purified with the Qiagen QIAquick PCR Purification Kit, eluted in 33 μl of 0.1X TE (1 mM Tris-HCl pH 8.0, 0.1 mM EDTA) and subject to ChIP-seq library preparation with the NEBNext Ultra II DNA Library Prep Kit for Illumina (NEB E7645) according to manufacturer instructions with a few modifications. 20 ng of ChIP input DNA (as measured by NanoDrop) and 25 μl of the immunoprecipitated DNA were used as starting material and the recommended reagents’ volumes were cut in half. The NEBNext Adaptor for Illumina was diluted 1:10 in Tris/NaCl, pH 8.0 (10 mM Tris-HCl pH 8.0, 10 mM NaCl) and the ligation step extended to 30 min. After ligation, a single purification step with 0.9X volumes of home-made SeraPure beads was performed, eluting DNA in 22 μl of 10 mM Tris-HCl pH 8.0. 20 μl of the eluted DNA were used for the library enrichment step, performed with the KAPA HotStart PCR kit (Roche Diagnostics KK2502) in 50 μl of total reaction volume (10 μl 5X KAPA buffer, 1.5 μl 10 mM dNTPs, 0.5 μl each of 10 μM primers, 1 μl KAPA polymerase, 16.5 μl nuclease-free water and 20 μl sample). Samples were enriched with nine PCR cycles [98°C, 45 s; (98°C, 15 s; 60°C, 10 s) × 9; 72°C, 1 min; 4°C, hold], purified with 0.9X volumes of SeraPure beads and eluted with 33 μl of 10 mM Tris-HCl pH 8.0. Library concentration, quality and fragment size were assessed by Qubit fluorometric quantification (Qubit dsDNA HS Assay Kit, Invitrogen Q32851), quantitative PCR (qPCR), and Fragment Analyzer. Multiplexed libraries were pooled and sent to MedGenome Inc. (Foster City, CA, USA) for sequencing on the Illumina NovaSeq 6000 (CTCF ChIP) or NovaSeq X Plus (Sp1 ChIP) sequencing platform (150-bp, paired-end reads).

### CUT&RUN

Cells were induced for 36 hours with 1 μg/ml doxycycline and simultaneously stained with 5 nM of JF549 Halo ligand and 5 nM of JFX650 SNAP ligand. After induction, cells were trypsinized and resuspended in medium containing 1 μg/ml doxycycline, then subjected to fluorescence-activated cell sorting (FACS) to select for transgene-expressing cells (induction levels typically ranged from ~10% to 50%). Sorted cells (between 90,000 and 280,000) were plated into individual wells of 6-well plates and cultured for an additional 36 hours in medium with 1 μg/ml doxycycline before initiation of the CUT&RUN protocol. CUT&RUN was performed as published (70, 71) with the following modifications and/or specifications. Digitonin was used at a final concentration of 0.02%. After the initial washes, cells were resuspended to 100 μl and transferred to 600-μl PCR tubes. We used 5 μl of BioMag Plus Concanavalin A beads (Fisher 86057-3) per sample, 1 μg of primary antibody (overnight incubation at 4°C), and 180 ng of in-house purified pAG-MNase (Addgene plasmid no. 123461). Chromatin was digested for 30 min on ice. After phenol/chloroform extraction, DNA was purified with 3X volumes of home-made SeraPure beads, binding for 1 hour at room temperature followed by two washes with 80% ethanol. Purified DNA was eluted in 30 μl 0.1X TE (1 mM Tris-HCl pH 8.0, 0.1 mM EDTA), 25 μl of which were used for library preparation, using NEBNext Ultra II DNA Library Prep Kit for Illumina (NEB E7645) according to manufacturer instructions modified for short fragments as described in (72). The NEBNext adaptor was diluted 1:20 in Tris/NaCl, pH 8.0 (10 mM Tris, 10 mM NaCl), and the library was enriched with 11 PCR cycles. After size selection, the library was eluted with 15 μl 10 mM Tris-HCl, pH 8.0. Library concentration was determined using Qubit fluorometric quantification (Qubit dsDNA HS Assay Kit, Invitrogen Q32851), library yield was assessed by qPCR, and fragment size distribution was evaluated on a Fragment Analyzer. Pooled libraries were sent to MedGenome Inc. (Foster City, CA, USA) for sequencing on the Illumina NovaSeq X Plus platform (150-bp, paired-end reads). See table S2 for a full list of all the samples and antibodies used in the CUT&RUN experiments.

### ChIP-seq and CUT&RUN data analysis

ChIP-seq and CUT&RUN raw fastq reads (48 libraries total) were first quality checked with FastQC (73) and then trimmed with Cutadapt (74) (v2.6) with the following parameters: -a AGATCGGAAGAGCACACGTCTGAACTCCAGTCA -A AGATCGGAAGAGCGTCGTGTAGGGAAAGAGTGT -m 15. Trimmed reads were aligned with Bowtie2 (75) (v2.3.5.1) with the following options: --local --very-sensitive-local --no-unal --no-mixed --no-discordant --phred33 -I 10 -X 700, using the hg38 human genome assembly. Bowtie2 output files were converted to .bam with samtools (v1.9). Bam files were sorted and indexed with samtools (v1.9), and duplicate reads removed with samtools (v1.9). Genome coverage files were generated with deepTools (76) bamCoverage (v3.5.4) with the following options: --binSize 10 --samFlagInclude 64 --normalizeUsing RPGC --effectiveGenomeSize 2913022398 --extendReads --ignoreDuplicates, using a published CUT&RUN hg38 blacklist file (77). To identify binding sites reproducible between biological replicates, we performed an irreproducible discovery rate (IDR) analysis (78). We first performed MACS3 (79) (v2.2.7.1) peak calling with a liberal P value cutoff (-p 1e-3) and --nolambda, merging control replicates to use as a control. We then sorted MACS3 peaks by their P value before running IDR (v2.0.4.2) with the following options: --input-file-type narrowPeak --rank p.value. For differential peak calling, we first combined biological replicates (bedGraph files) with MACS3 cmbreps (--method mean). We then used the combined bedGraph outputs for differential peak calling with MACS3 bdgdiff with the following options: --cutoff 1.0 --depth1 1.0 --depth2 1.0 -l 120 -g 60, again using merged control replicates as a control. Only peaks with an IDR score ≤ 0.1 were considered for further analysis. To evaluate peak overlap between different conditions we used bedops (v2.4.39). Heatmaps were generated with deepTools (v3.5.4) computeMatrix and plotHeatmap tools. Spearman correlation values were computed with deepTools (v3.5.4) multiBigWigSummary and plotCorrelation tools. Read numbers are provided in table S2. Peak numbers are provided in table S3.

### Antibodies

Antibodies used in this study are listed in table S4.

## Supplementary Material

data_s1

table_s1

table_s2

table_s3

table_s4

Abidi2026_SupplementaryText

## Data Availability

Sequencing data have been deposited to the Gene Expression Omnibus (GEO) under the accession no. GSE300856 (ChIP-seq: SubSeries GSE300853; CUT&RUN: SubSeries GSE300854). Single-molecule trajectories for all SMT and PAPA results and FRAP movies are available through Zenodo (80). Table S4 lists all the DNA constructs used to generate the cell lines described in this study. DNA sequences are available as supplementary files. All the cell lines used in this study are available upon request. Data analysis scripts are available through Zenodo (80).
