# Supplementary material for: Unstructured transcription factor interactions enable emergent specificity
Source: Science. Author manuscript; Available in PMC 2026 Sep 16. (PMC13581189; doi:10.1126/science.aeb6487)
Supplement: Supplementary file 1 [file NIHMS2192036-supplement-Abidi2026_SupplementaryText.pdf]

Supplementary Materials for

Unstructured transcription factor interactions enable emergent specificity

Abrar A. Abidi, Claudia Cattoglio, Natalie N. Tang, Vinson B. Fan, Gina M. Dailey, Amir D. Hay, Prasanthi Kunamaneni, Daniel E. Milkie, Xavier Darzacq, Eric Betzig, Robert Tjian, Thomas G. W. Graham

Corresponding authors: jmlim@berkeley.edu (R.T.), tgraha22@jhu.edu (T.G.W.G.)

The PDF file includes:

Figs. S1 to S21

Other Supplementary Materials for this manuscript include the following:

Tables S1 to S4
Data S1

Fig. S1.

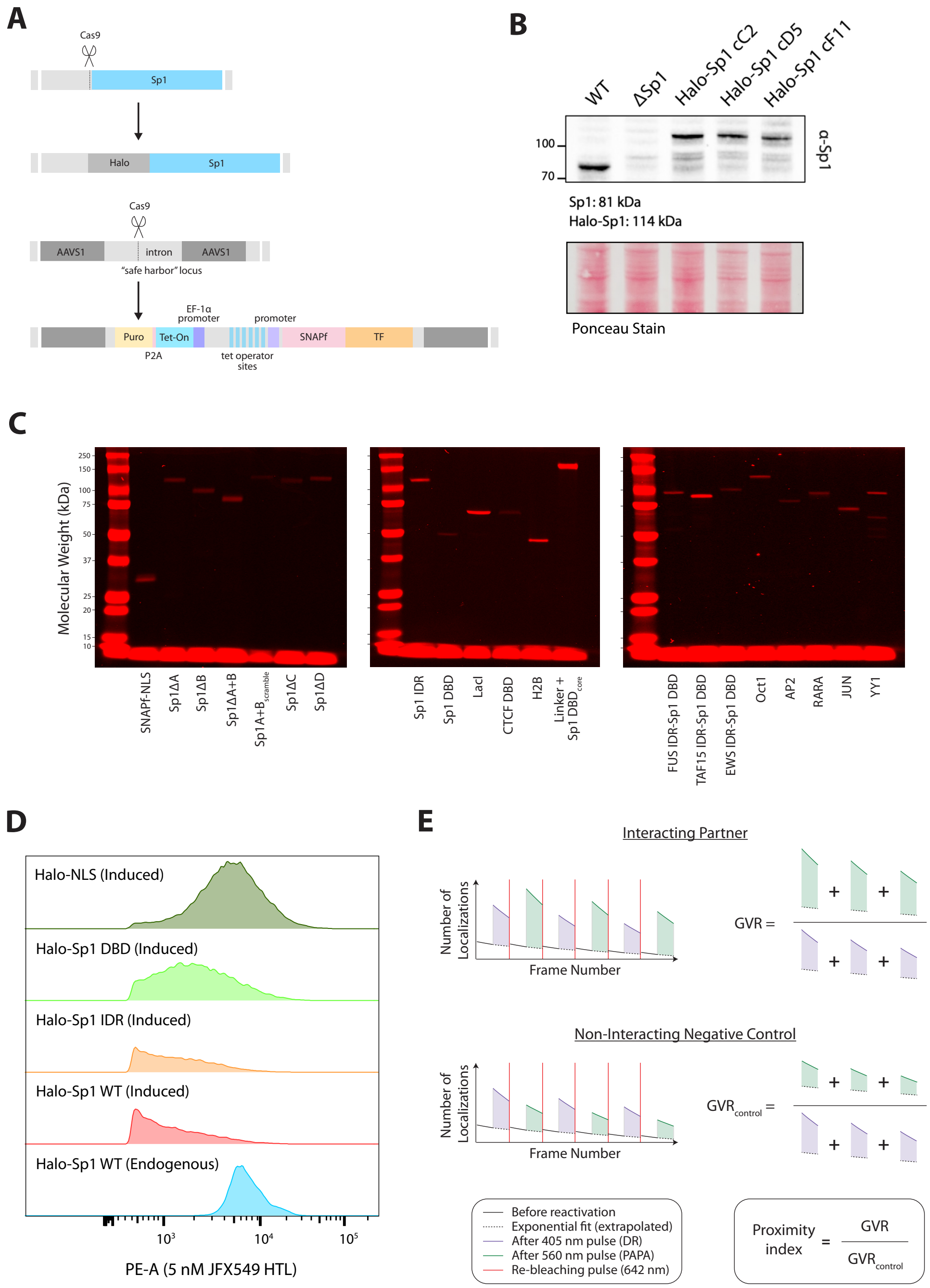

Engineering strategy for Halo and SNAPf fusions enables PAPA measurements and proximity index calculation at sub-physiological expression levels. (A) Schematic of genome engineering strategy. A HaloTag was inserted at the N-terminus of the endogenous Sp1 locus in U2OS cells using CRISPR-Cas9, generating an endogenously expressed Halo-Sp1 fusion. Separately, SNAPf-TF fusion constructs driven by a doxycycline-inducible promoter were integrated at the *AAVS1* "safe harbor" locus for tunable expression of Sp1 variants. **(B)** Western blot of cell lysates from wild-type (WT), Sp1 knockout (ΔSp1), and Halo-Sp1 knock-in clones (cC2, cD5, cF11). Blot was probed with an anti-Sp1 antibody, confirming loss of endogenous

Sp1 in the ΔSp1 line and the expected size shift of the Halo-Sp1 fusion protein (~114 kDa) relative to native Sp1 (~81 kDa). The lower panel shows Ponceau staining of total protein as a loading control. **(C)** SDS-PAGE gel of lysates prepared from cell lines expressing inducible SNAPf fusion proteins, stained with JFX650 SNAPf ligand. Molecular weight markers (kDa) are indicated on the left, and the identities of labeled proteins are indicated below. Ladder bands are saturated in the JFX650 channel. **(D)** Flow cytometry quantification of expression levels for inducible Halo-fused constructs (Sp1 WT, Sp1 IDR, Sp1 DBD, and NLS) integrated at *AAVS1* in the ΔSp1 background. Expression levels are compared to endogenously expressed Halo-Sp1, enabling matched single-molecule imaging at comparable nuclear concentrations. **(E)** Calculation of the proximity index. The total number of localizations in each frame is counted before (black curves) and after violet and green light pulses. Red light pulses between cycles (red lines) bleach or re-shelve receiver fluorophores. The number of green- and violet-reactivated localizations is calculated as the difference between the observed number of localizations and the anticipated baseline based on an exponential fit of the preceding pre-activation period. The green-to-violet ratio (GVR) is determined for a pair of candidate interacting partners (top row) and a matched non-interacting negative control (bottom row). The proximity index is defined as the ratio between these two GVRs. A proximity index of 1 corresponds to nonspecific background levels of reactivation.

Fig. S2.

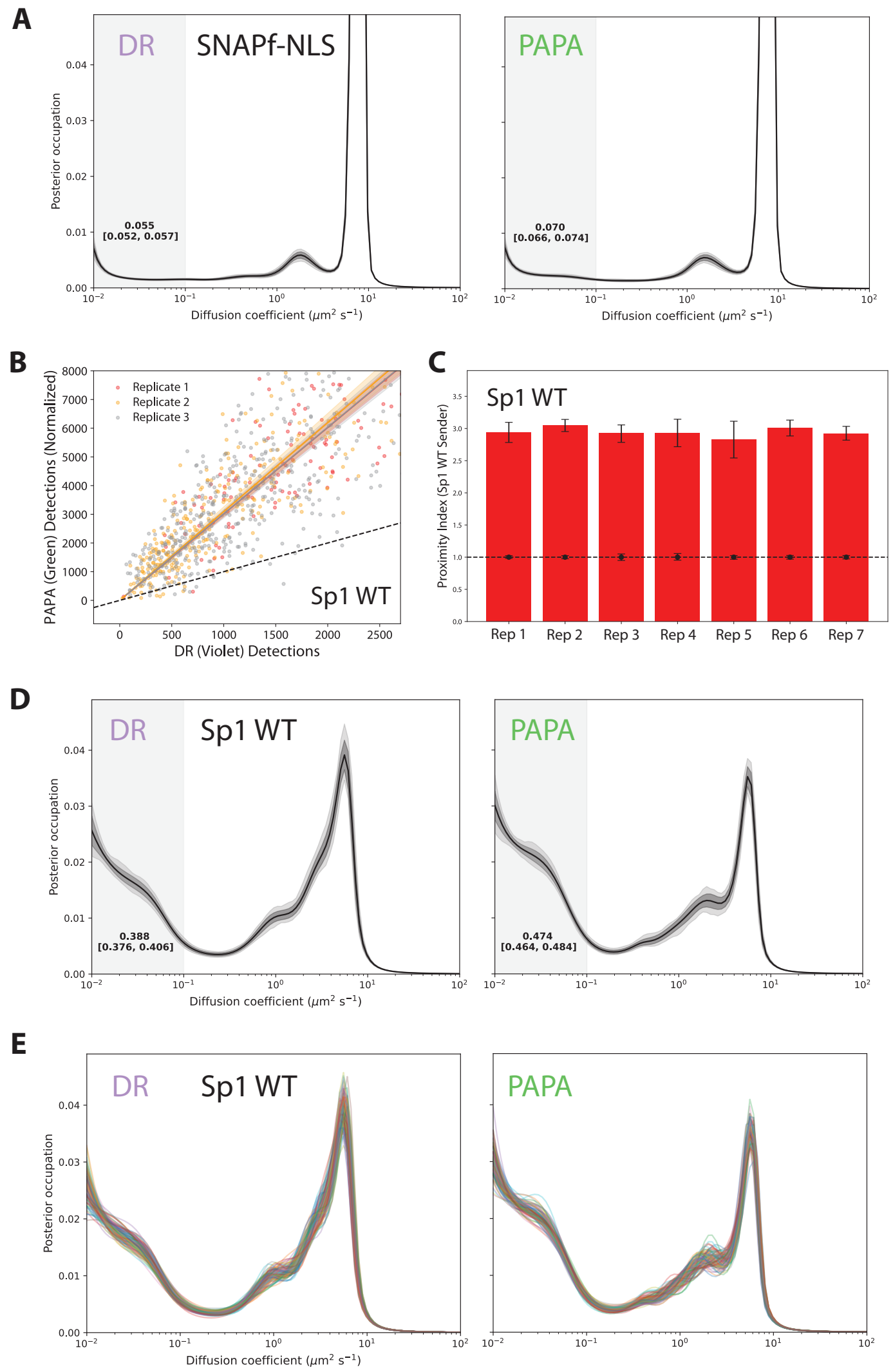

Reproducibility of PAPA measurements for wild-type Sp1/Sp1 interaction.
(A) Bootstrapped diffusion coefficient distributions of non-interacting SNAPf-NLS control receiver-labeled molecules reactivated by DR or PAPA. Black lines show posterior mean diffusion coefficient distributions; light gray shading indicates the 95% confidence interval estimated by bootstrapping across replicates, and dark gray shading indicates the standard deviation across 96 bootstrapped replicates. Values reported on each plot indicate the bound fraction ($D < 0.1$ μm²/s) with the 95% confidence interval shown in square brackets. **(B)** Scatter plot of normalized green-reactivated (PAPA) versus violet-reactivated (DR) detections at the single-cell level for three independent replicates of the Sp1 sender-Sp1 receiver PAPA experiment. Solid line indicates linear fit; dotted line indicates a slope of 1 (no interaction). Detections were normalized to the negative control so that a slope of 1 corresponds to no interaction. **(C)** Proximity index values for Sp1/Sp1 across seven biological replicates, showing high reproducibility. Error bars represent 95% confidence intervals. **(D)** Diffusion coefficient distributions of receiver-labeled Sp1 under DR and PAPA conditions, plotted as in (A). **(E)** Individual bootstrap replicates used to generate confidence intervals in (D), illustrating the consistency of diffusion profiles across iterations.

Fig. S3.

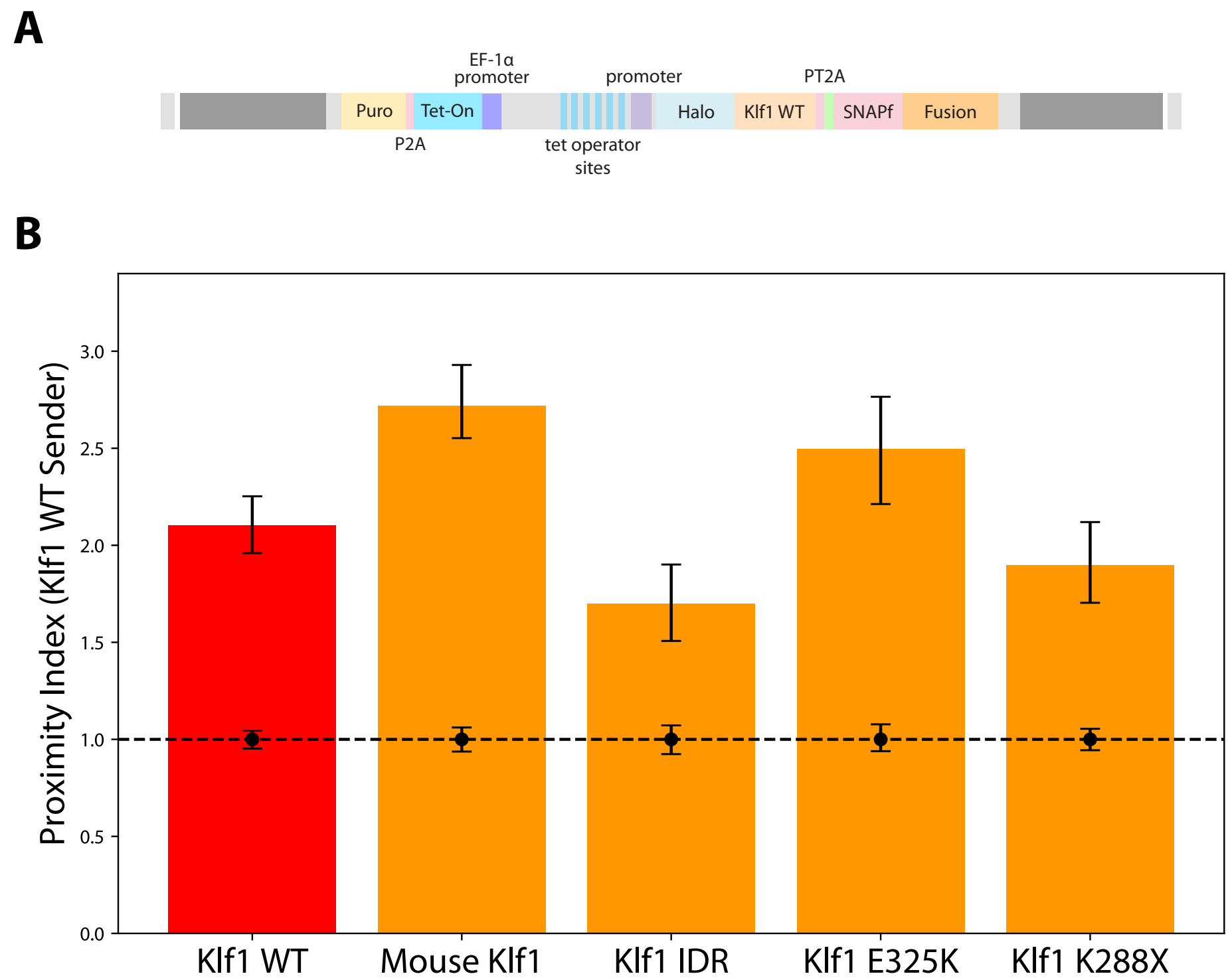

Klf1 constructs. (A) Schematic of the inducible bicistronic construct used to co-express Halo-Klf1 WT and SNAPf-fusion proteins. **(B)** Proximity index measurements for sender-labeled human Klf1 paired with receiver-labeled full-length human Klf1 (WT), mouse Klf1, the isolated N-terminal Klf1 IDR, and two disease-associated variants (E325K and K288X). Bars represent mean proximity index; error bars indicate 95% bootstrap confidence intervals.

Fig. S4.

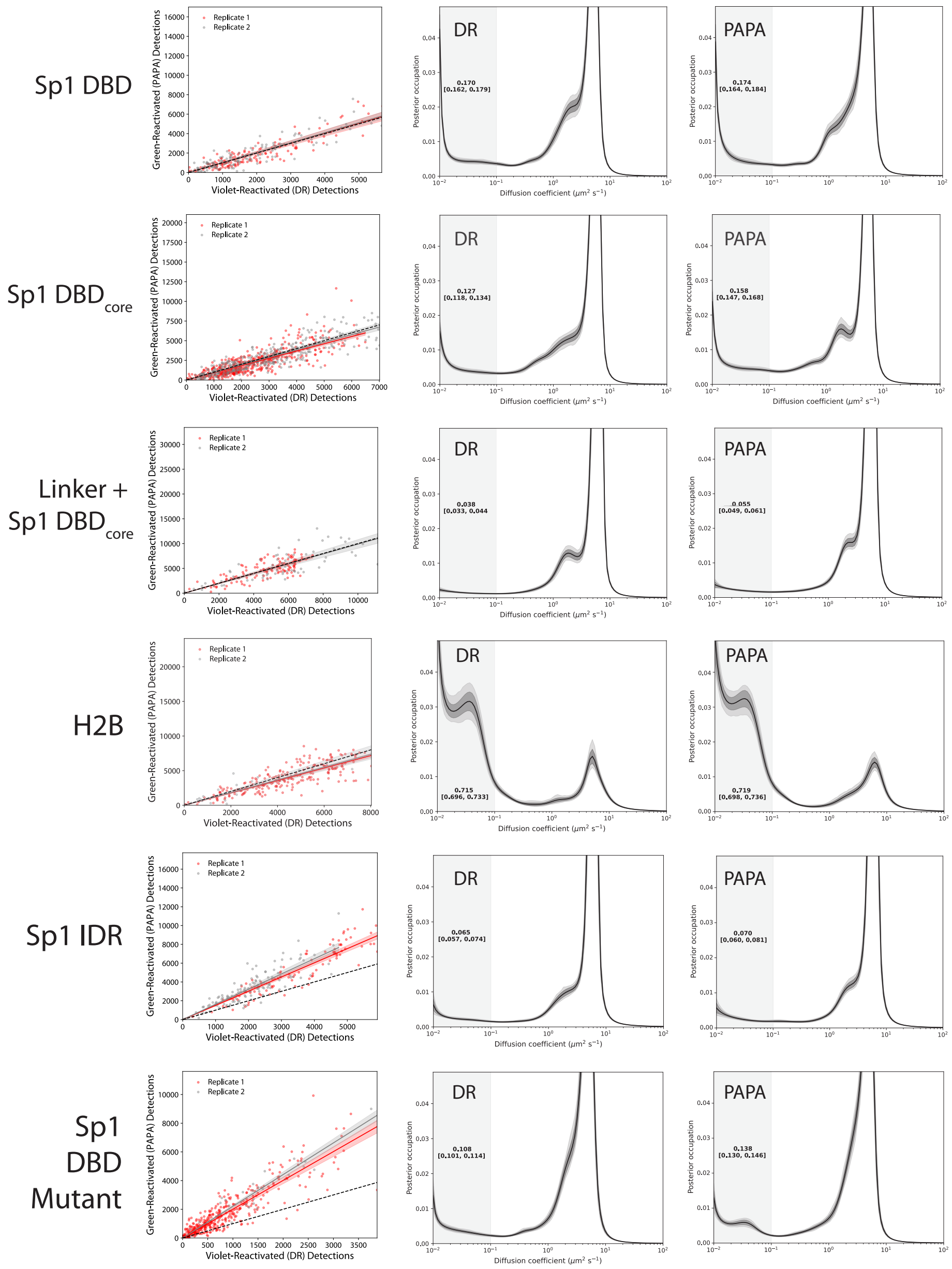

Quantitative analysis of isolated DBD and IDR variants using PAPA and single molecule tracking. For each receiver-labeled construct listed on the left (Sp1 DBD, Sp1 DBD_{core}, Linker + Sp1 DBD_{core}, H2B, Sp1 IDR, and Sp1 DBD Mutant): (Left) Scatter plots showing normalized green-reactivated (PAPA) versus violet-reactivated (DR) detections at the single-cell level across two biological replicates. Solid lines indicate linear regression fits; dashed lines indicate a slope of 1 (no interaction). Detections were normalized to the negative control so that a slope of 1 corresponds to no interaction. (Middle and Right) Bootstrapped diffusion coefficient distributions of receiver-labeled molecules reactivated by DR or PAPA. Black lines show posterior mean diffusion coefficient distributions; light gray shading indicates the 95% confidence interval estimated by bootstrapping across replicates, and dark gray shading indicates the standard deviation across 96 bootstrapped replicates. Values reported on each plot indicate the bound fraction ($D < 0.1$ μm²/s) with the 95% confidence interval shown in square brackets.

Fig. S5.

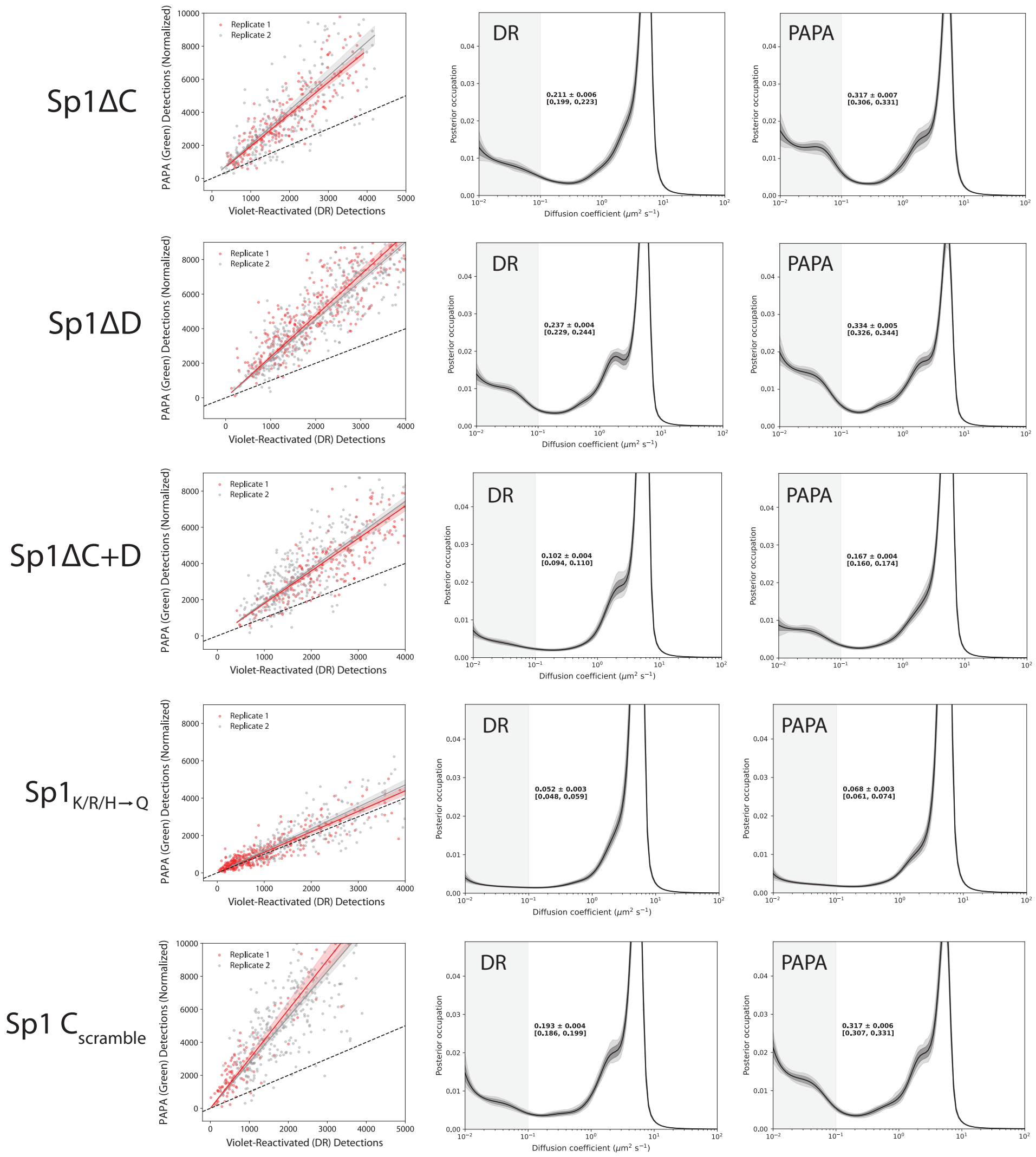

Quantitative analysis of basic residue mutants using PAPA and single-molecule tracking. For each receiver-labeled construct listed on the left (Sp1ΔC, Sp1ΔD, Sp1ΔC+D, $Sp1_{K/R/H\rightarrow Q}$, and $Sp1C_{scramble}$): (Left) Scatter plots showing normalized green-reactivated (PAPA) versus violet-reactivated (DR) detections at the single-cell level across two biological replicates. Solid lines indicate linear regression fits; dashed lines indicate a slope of 1 (no interaction). Detections were normalized to the negative control so that a slope of 1 corresponds to no interaction. (Middle and Right) Bootstrapped diffusion coefficient distributions of receiver-labeled molecules reactivated by DR or PAPA. Black lines show posterior mean diffusion coefficient distributions; light gray shading indicates the 95% confidence interval estimated by bootstrapping across replicates, and dark gray shading indicates the standard deviation across 96 bootstrapped replicates. Values reported on each plot indicate the bound fraction (D < 0.1 μm²/s) with the 95% confidence interval shown in square brackets.

Fig. S6.

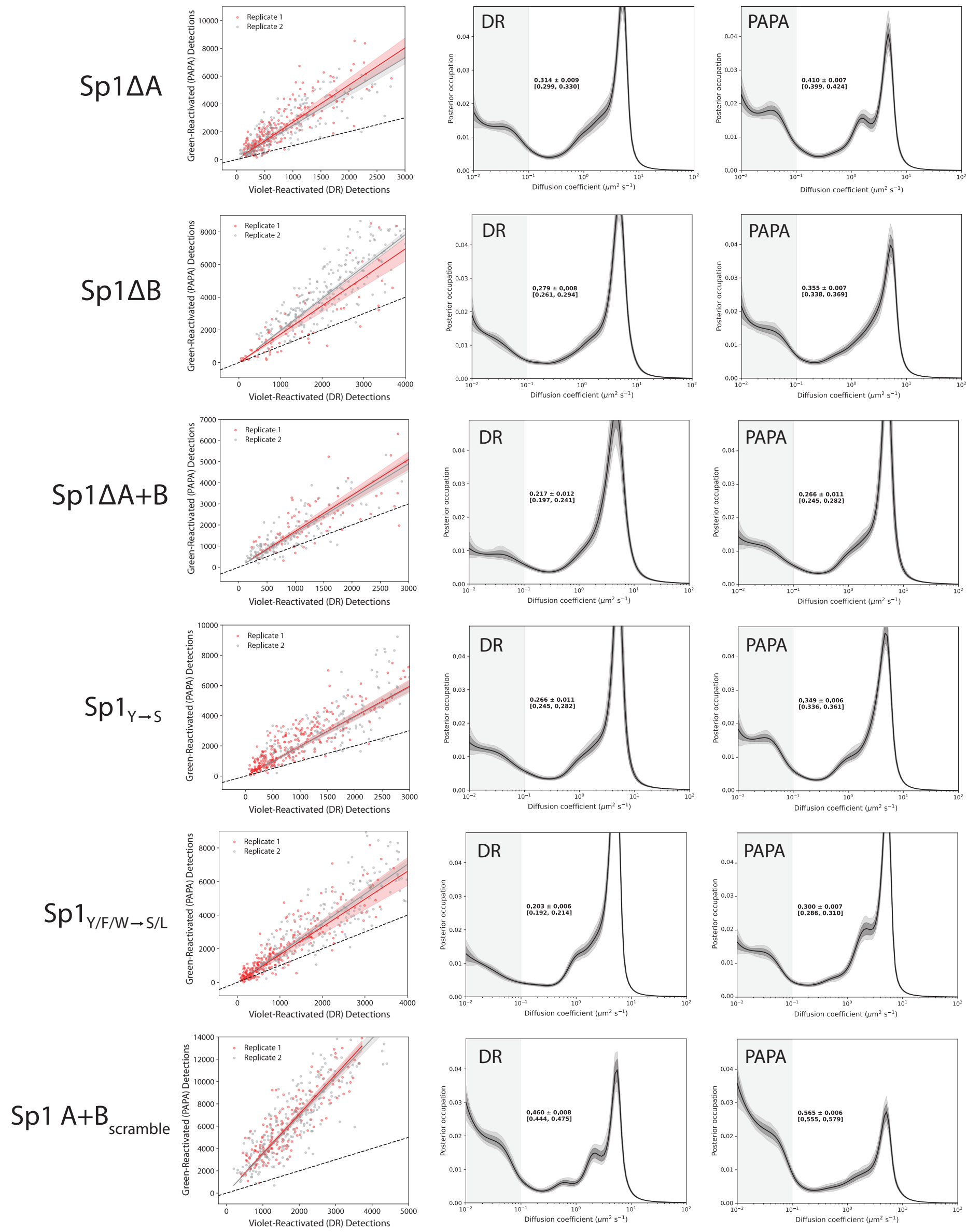

Quantitative analysis of Sp1 aromatic residue mutants using PAPA and single-molecule tracking. For each receiver-labeled construct listed on the left (Sp1ΔA, Sp1ΔB, Sp1ΔA+B, $Sp1_{Y\rightarrow S}$, $Sp1_{Y/F/W\rightarrow S/L}$, and $Sp1A{+}B_{scramble}$): (Left) Scatter plots showing normalized green-reactivated (PAPA) versus violet-reactivated (DR) detections at the single-cell level across two biological replicates. Solid lines indicate linear regression fits; dashed lines indicate a slope of 1 (no interaction). Detections were normalized to the negative control so that a slope of 1 corresponds to no interaction. (Middle and Right) Bootstrapped diffusion coefficient distributions of receiver-labeled molecules reactivated by DR or PAPA. Black lines show posterior mean diffusion coefficient distributions; light gray shading indicates the 95% confidence interval estimated by bootstrapping across replicates, and dark gray shading indicates the standard deviation across 96 bootstrapped replicates. Values reported on each plot indicate the bound fraction ($D < 0.1$ μm²/s) with the 95% confidence interval shown in square brackets.

Fig. S7.

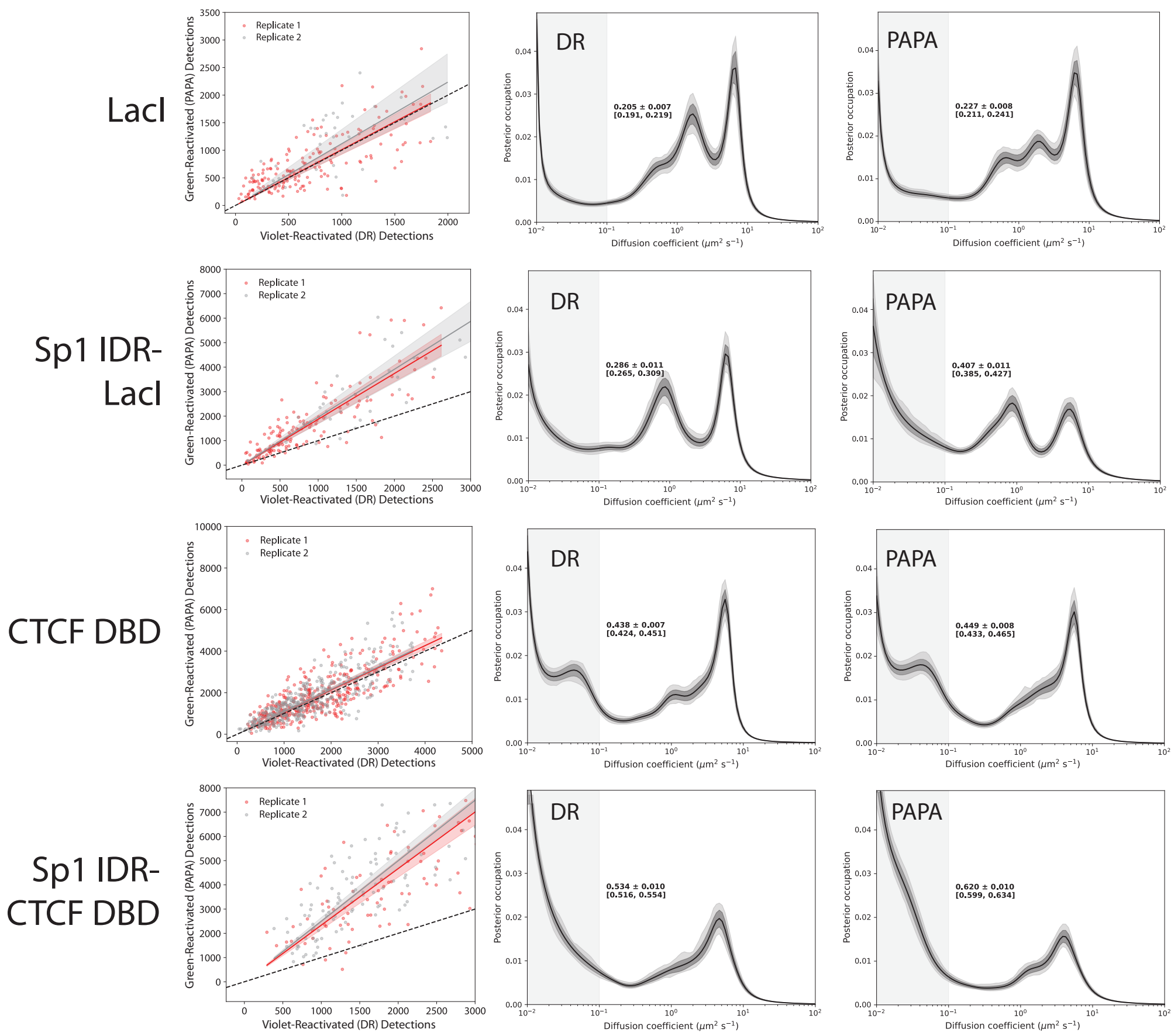

Quantitative analysis of LacI and CTCF DBD swap variants using PAPA and single-molecule tracking. For each receiver-labeled construct listed on the left (LacI, Sp1 IDR-LacI, CTCF DBD, and Sp1 IDR-CTCF DBD): (Left) Scatter plots showing normalized green-reactivated (PAPA) versus violet-reactivated (DR) detections at the single-cell level across two biological replicates. Solid lines indicate linear regression fits; dashed lines indicate a slope of 1 (no interaction). Detections were normalized to the negative control so that a slope of 1 corresponds to no interaction. (Middle and Right) Bootstrapped diffusion coefficient distributions of receiver-labeled molecules reactivated by DR or PAPA. Black lines show posterior mean diffusion coefficient distributions; light gray shading indicates the 95% confidence interval estimated by bootstrapping across replicates, and dark gray shading indicates the standard deviation across 96 bootstrapped replicates. Values reported on each plot indicate the bound fraction ($D < 0.1$ μm²/s) with the 95% confidence interval shown in square brackets.

Fig. S8.

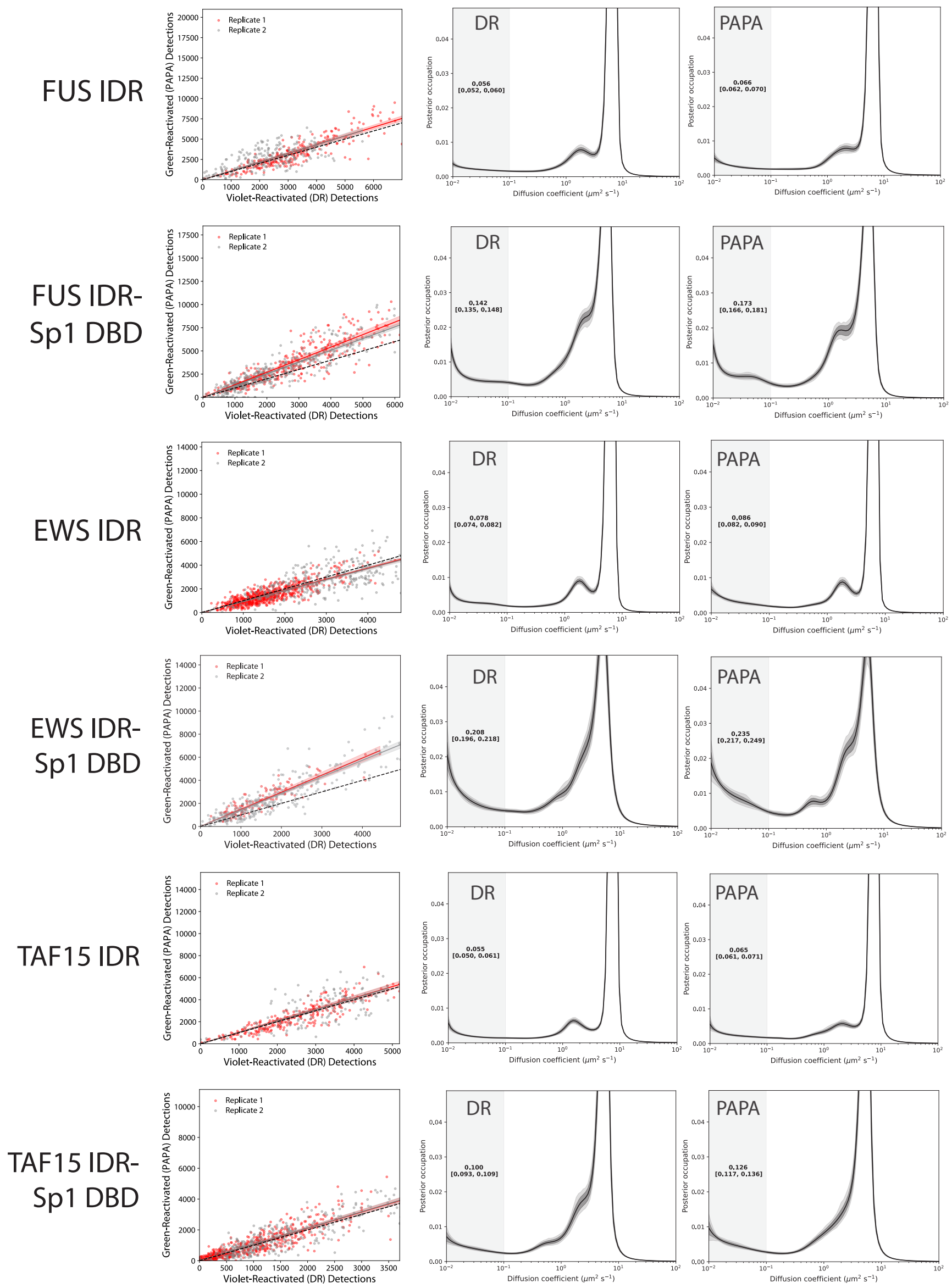

Quantitative analysis of FET family IDR swap variants using PAPA and single-molecule tracking. For each receiver-labeled construct listed on the left (FUS IDR, FUS IDR-Sp1 DBD, EWS IDR, EWS IDR-Sp1 DBD, TAF15 IDR, TAF15 IDR-Sp1 DBD): (Left) Scatter plots showing normalized green-reactivated (PAPA) versus violet-reactivated (DR) detections at the single-cell level across two biological replicates. Solid lines indicate linear regression fits; dashed lines indicate a slope of 1 (no interaction). Detections were normalized to the negative control so that a slope of 1 corresponds to no interaction. (Middle and Right) Bootstrapped diffusion coefficient distributions of receiver-labeled molecules reactivated by DR or PAPA. Black lines show posterior mean diffusion coefficient distributions; light gray shading indicates the 95% confidence interval estimated by bootstrapping across replicates, and dark gray shading indicates the standard deviation across 96 bootstrapped replicates. Values reported on each plot indicate the bound fraction ($D < 0.1$ μm²/s) with the 95% confidence interval shown in square brackets.

Fig. S9.

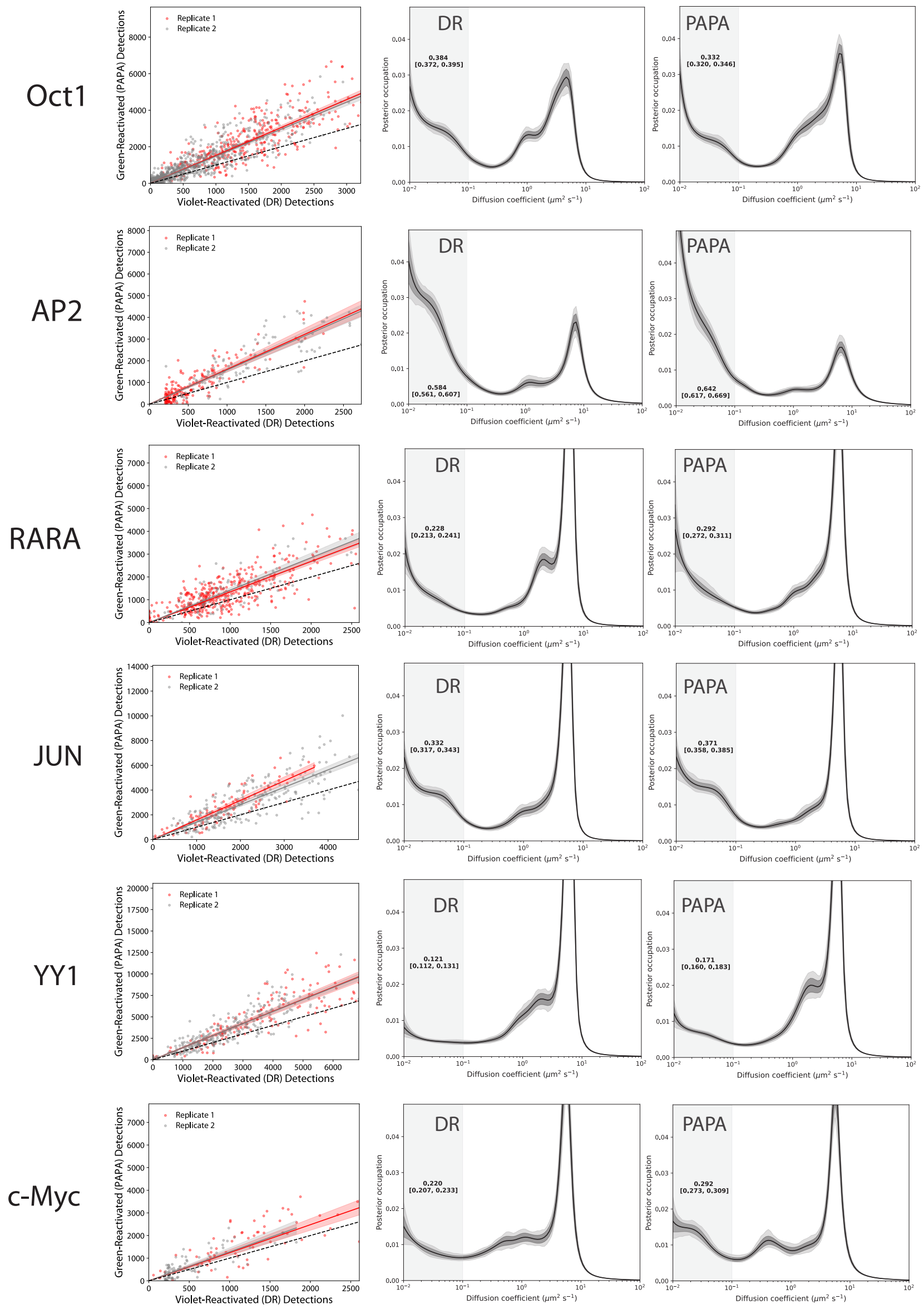

Quantitative analysis of Sp1 heterotypic partner TFs using PAPA and single-molecule tracking. For each receiver-labeled full-length transcription factor listed on the left (Oct1, AP2, RARA, JUN, YY1, and c-Myc): (Left) Scatter plots showing normalized green-reactivated (PAPA) versus violet-reactivated (DR) detections at the single-cell level across two biological replicates. Solid lines indicate linear regression fits; dashed lines indicate a slope of 1 (no interaction). Detections were normalized to the negative control so that a slope of 1 corresponds to no interaction. (Middle and Right) Bootstrapped diffusion coefficient distributions of receiver-labeled molecules reactivated by DR or PAPA. Black lines show posterior mean diffusion coefficient distributions; light gray shading indicates the 95% confidence interval estimated by bootstrapping across replicates, and dark gray shading indicates the standard deviation across 96 bootstrapped replicates. Values reported on each plot indicate the bound fraction (D < 0.1 μm²/s) with the 95% confidence interval shown in square brackets.

Fig. S10.

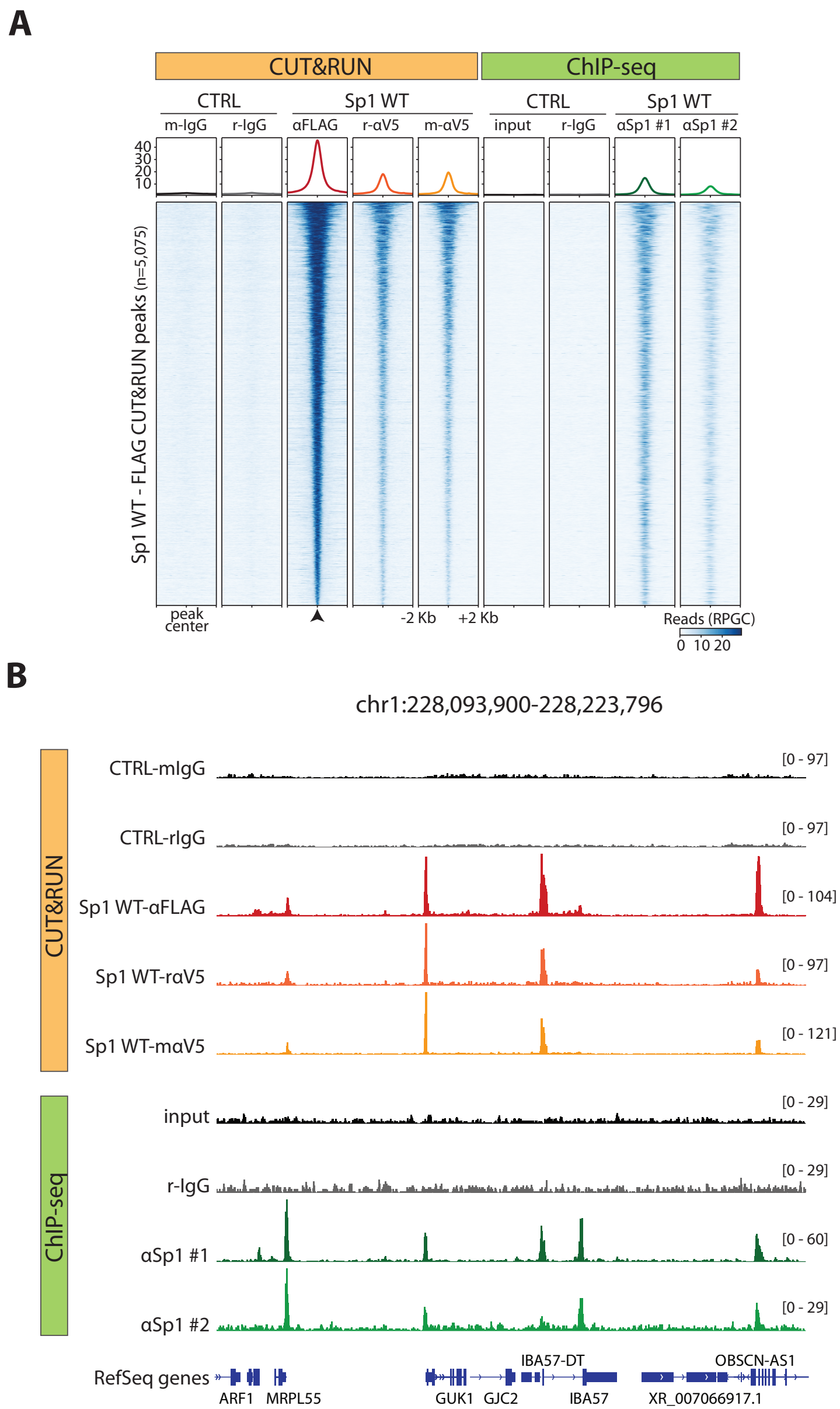

CUT&RUN and ChIP-seq profiling of Sp1 WT-FLAG binding across the genome. (A) Heatmap showing CUT&RUN and ChIP-seq read densities (Reads Per Genomic Content) ±2 kb around the center of 5,075 reproducible FLAG peaks identified by CUT&RUN in the inducible SNAPf-Sp1 WT-FLAG line (IDR score ≤ 0.1). Samples include CUT&RUN (orange) and ChIP-seq (green) data for the indicated antibodies in the parental U2OS V5-Halo-Sp1 knock-in line (CTRL) and the corresponding SNAPf-Sp1 WT-FLAG inducible line. Antibody prefixes denote host species (m = mouse, r = rabbit). The arrowhead marks the peak center used to align the heatmap rows, which are sorted top to bottom by descending peak intensity. **(B)** Genome browser tracks of representative CUT&RUN and ChIP-seq signal at a representative locus (chr1:228,093,900-228,223,796), corresponding to the peaks in (A). Antibodies and sample types are indicated on the left. Data range for each track is shown in brackets; control tracks are scaled to the lowest range to enable comparison. Gene annotations (RefSeq) are shown at the bottom.

Fig. S11.

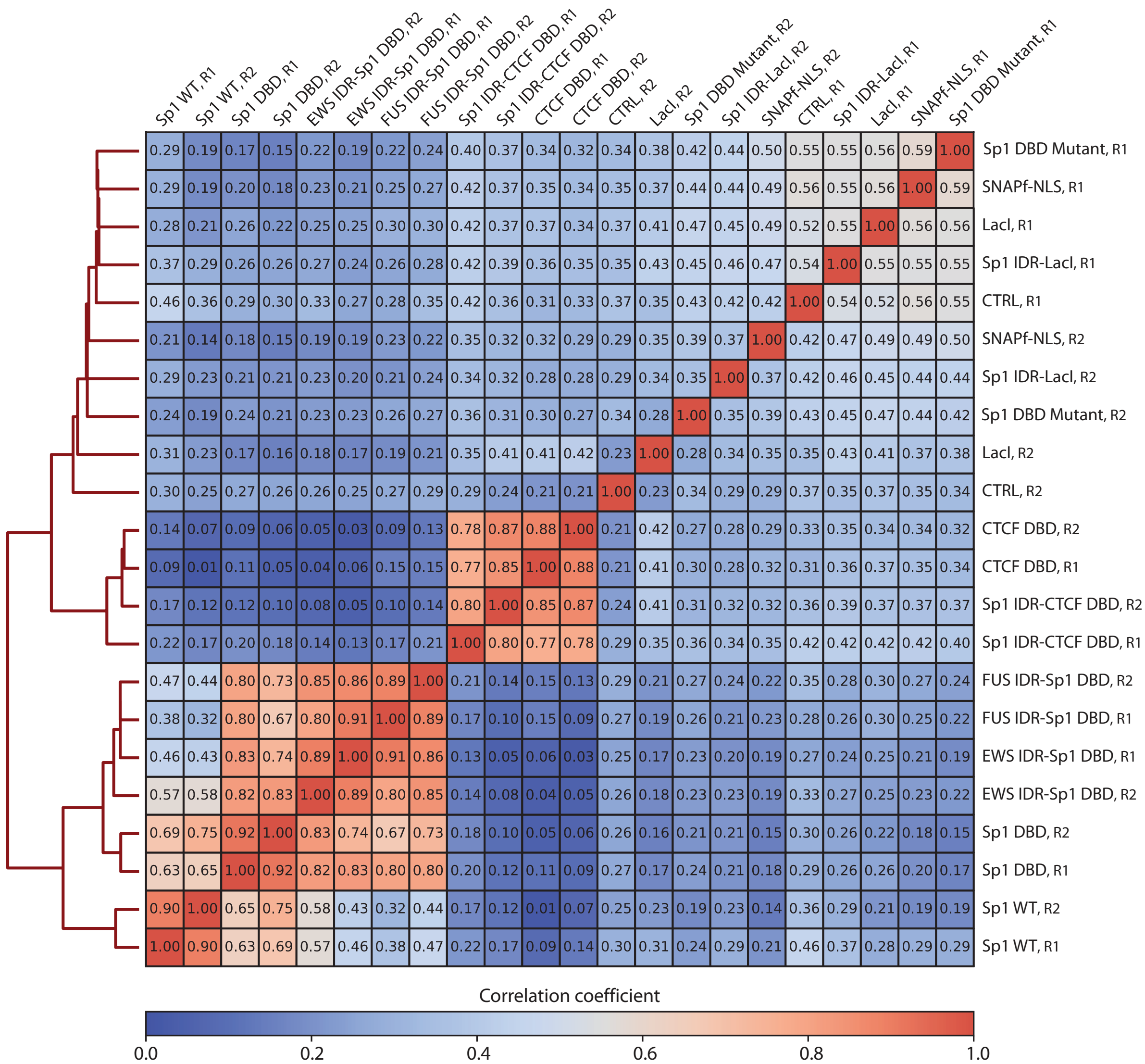

Spearman correlation heatmap of genome-wide FLAG CUT&RUN profiles. Heatmap showing pairwise Spearman correlation coefficients between genome-wide CUT&RUN coverage tracks (±2 kb around 5,075 reproducible FLAG peaks, IDR score ≤ 0.1) across two biological replicates (R1, R2) for each sample. This expanded version of the heatmap shown in Fig. 5G includes the parental U2OS V5-Halo-Sp1 knock-in line (CTRL), the inducible SNAPf-Sp1 WT-FLAG line, and various other constructs: mutant Sp1, SNAPf-NLS, LacI, Sp1 IDR-LacI, Sp1 IDR-CTCF DBD, FUS IDR-Sp1 DBD, EWS IDR-Sp1 DBD, and others.

Fig. S12.

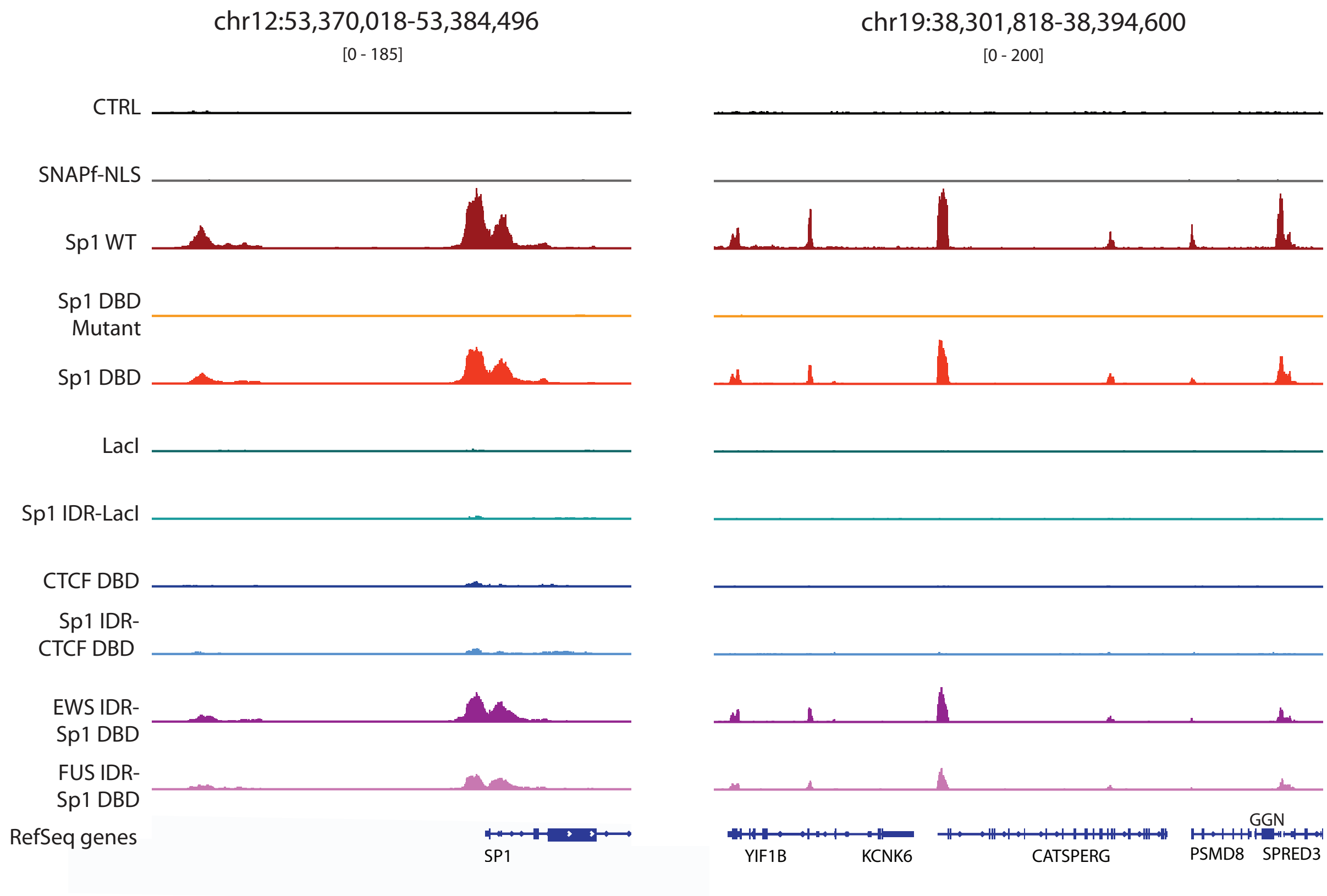

Genome browser tracks of FLAG CUT&RUN signal across Sp1 and chimeric constructs. Representative IGV tracks showing FLAG CUT&RUN signal at two genomic loci—on chromosomes 12 (left) and 19 (right)—for the samples described in Fig. 5. Tracks include the parental CTRL line, SNAPf-NLS, Sp1 WT, Sp1 DBD Mutant, isolated Sp1 DBD, LacI, Sp1 IDR-LacI, CTCF DBD, Sp1 IDR-CTCF DBD, EWS IDR-Sp1 DBD, and FUS IDR-Sp1 DBD. Genomic coordinates are listed at the top, with the data range for each set of tracks specified in square brackets. Signal is scaled uniformly across samples at each locus to highlight relative enrichment.

Fig. S13.

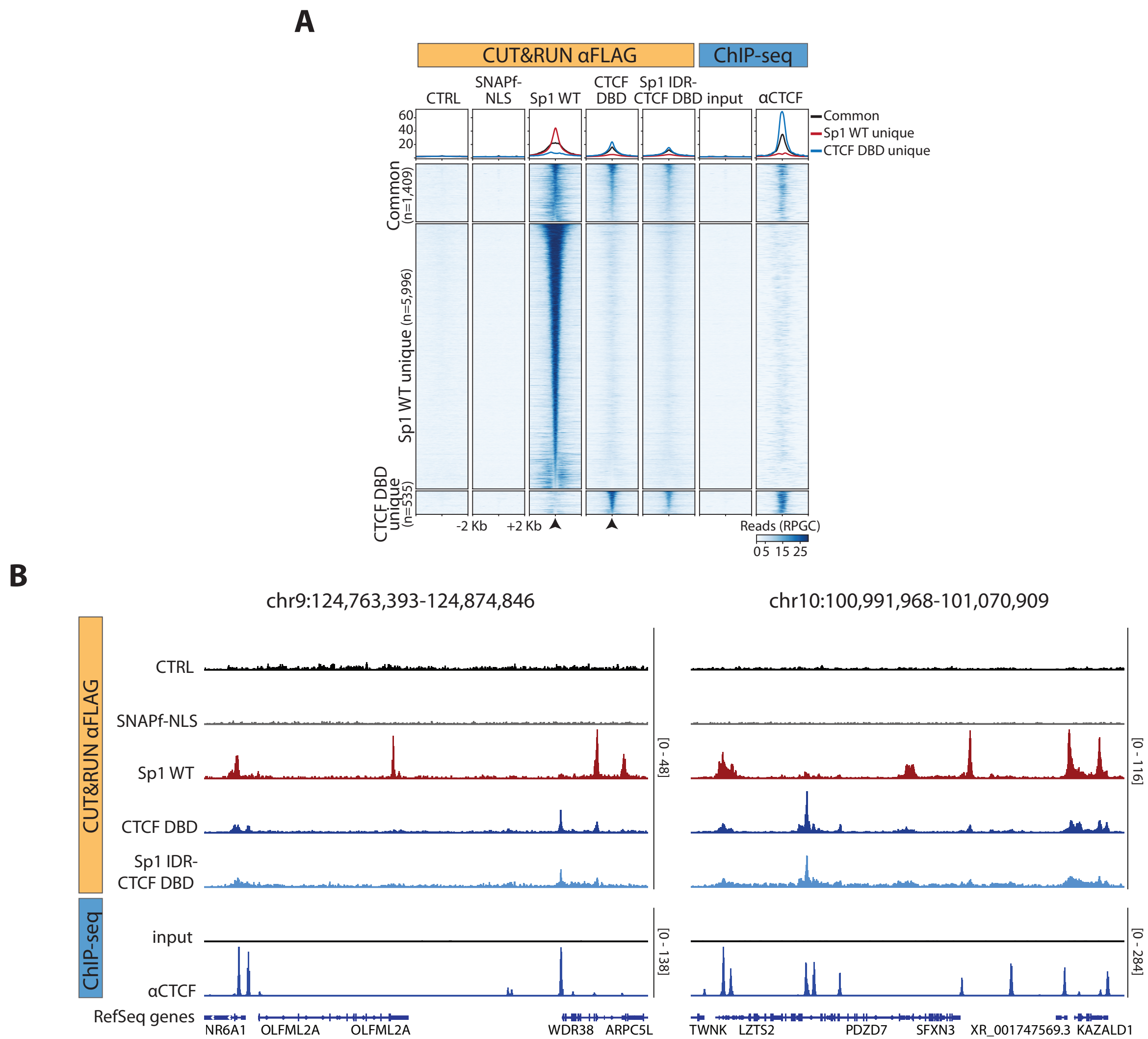

Comparison of CUT&RUN and ChIP-seq signal between Sp1 WT and CTCF DBD chimeras. (A) Heatmap of FLAG CUT&RUN and CTCF ChIP-seq signal (Reads Per Genomic Content) ±2 kb around the center of 10,163 differential FLAG peaks identified in the inducible SNAPf-Sp1 WT-FLAG versus SNAPf-Sp1 IDR-CTCF DBD lines. Tracks include CUT&RUN profiles from the parental control (CTRL) line and the indicated inducible constructs, along with endogenous CTCF ChIP-seq signal from U2OS cells. The heatmap is sorted separately for peaks common to both lines, peaks unique to Sp1 WT (top), and peaks unique to CTCF DBD (bottom), as indicated on the left. Arrowheads denote the samples used to sort each set. Line plots above show the average signal for each condition across the indicated peaks. **(B)** Representative genome browser tracks showing FLAG CUT&RUN and CTCF ChIP-seq signal at two genomic loci on chromosomes 9 and 10. Genomic coordinates are shown at top, and data ranges for each track group are indicated in square brackets. Signal is scaled to the highest value within each group.

Fig. S14.

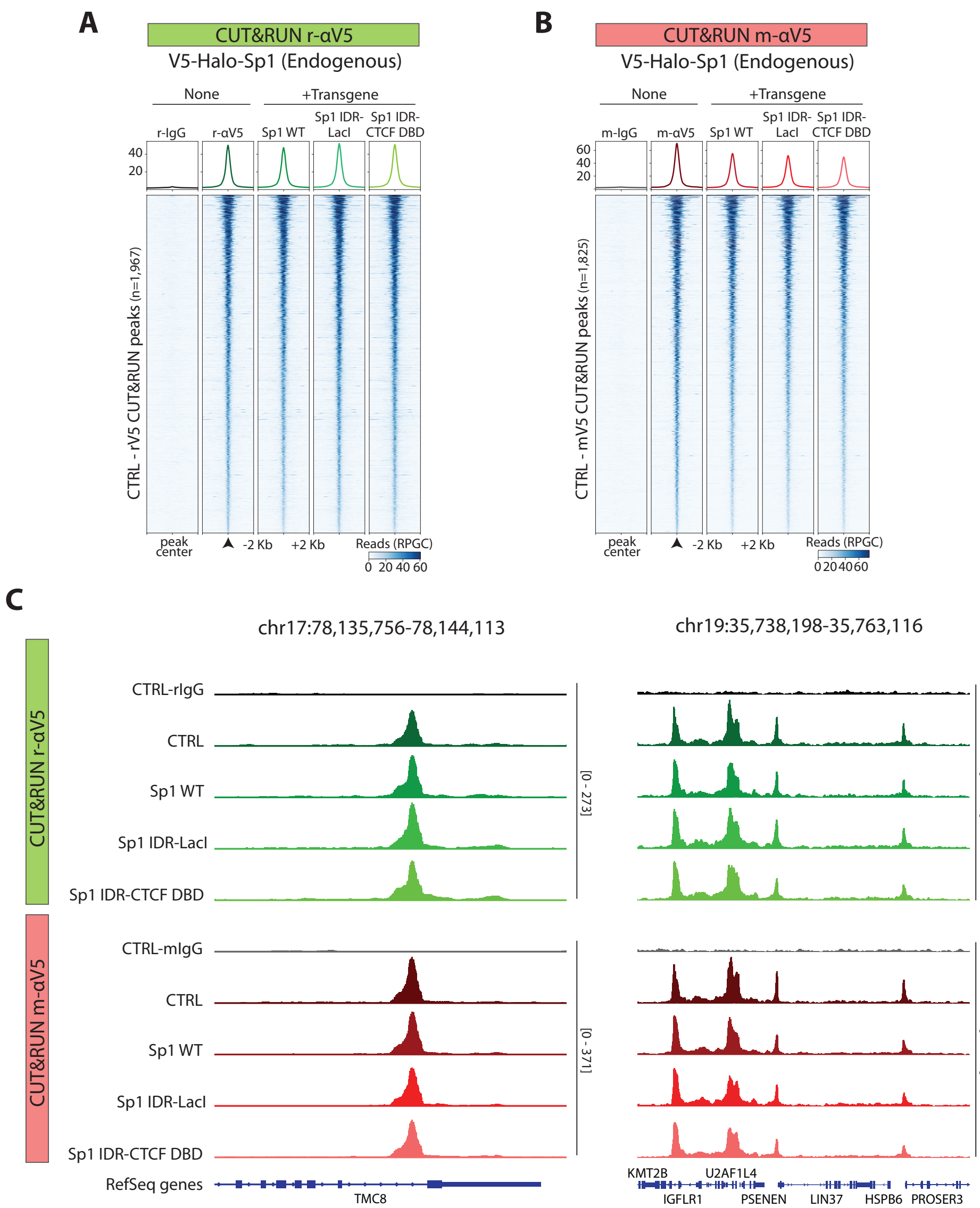

CUT&RUN profiling of Sp1 in chimeric construct background using rabbit and mouse V5 antibodies. (A) Heatmap of FLAG CUT&RUN signal (Reads Per Genomic Content) ±2 kb around the center of 1,967 reproducible peaks (IDR score ≤ 0.1) identified using the rabbit anti-V5 antibody (rV5) in the parental U2OS V5-Halo-Sp1 knock-in line (CTRL). Shown are CUT&RUN profiles for the CTRL line and the indicated inducible lines expressing Sp1 WT or chimeras containing the N-terminal Sp1 IDR fused to either LacI or the CTCF DNA-binding domain (DBD). Heatmap rows are sorted by peak intensity in the CTRL sample (arrowhead). Line plots above display the mean signal across all peaks. **(B)** Same as (A) but using the mouse anti-V5 antibody (mV5), which yielded 1,825 reproducible peaks in the CTRL line. **(C)** IGV tracks of representative CUT&RUN signal from rV5 (left) and mV5 (right) datasets at genomic loci on chromosomes 17 and 19. Genomic coordinates are listed at the top. Data ranges for each track group are indicated in square brackets. Tracks are scaled to the highest signal within each panel.

Fig. S15.

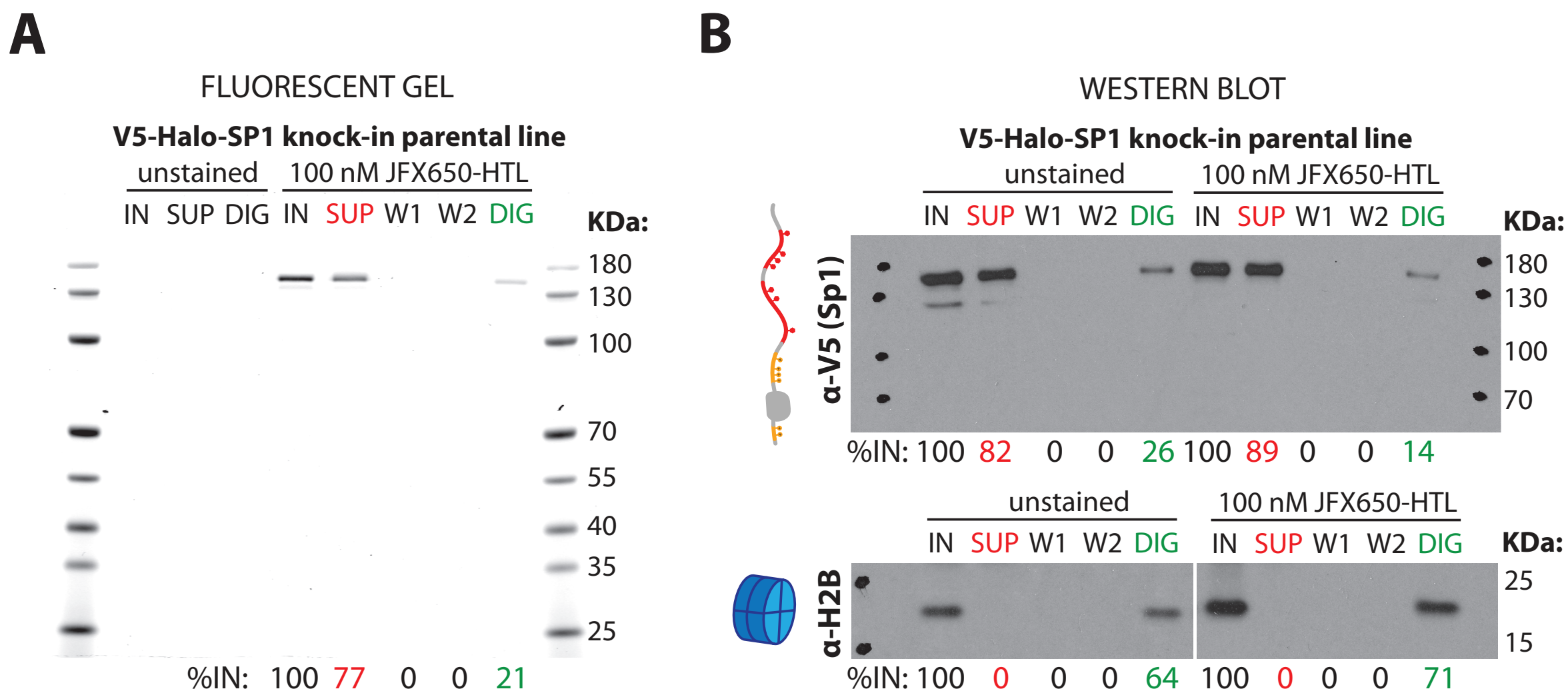

Nuclear depletion of Sp1 from CUT&RUN permeabilization. (A) Fluorescent gel showing total levels of Sp1 (input, IN) in cells subject to CUT&RUN and substantial protein loss (77% of input) in the routinely discarded supernatant (SUP, red) after overnight incubation with antibodies. Only ~20% of the initial Sp1 protein is retained in the MNase digested sample (DIG, green). Cells were stained with JFX650 HaloTag ligand (HTL) prior to the mock CUT&RUN experiment and SDS-PAGE. Unstained cells were used as control for autofluorescence. W1 and W2: consecutive washes routinely performed prior to MNase addition. **(B)** Western blot of the same samples in (A) probing for Sp1 protein loss (top) and H2B, which is instead retained in the MNase digested material despite the digitonin permeabilization.

Fig. S16.

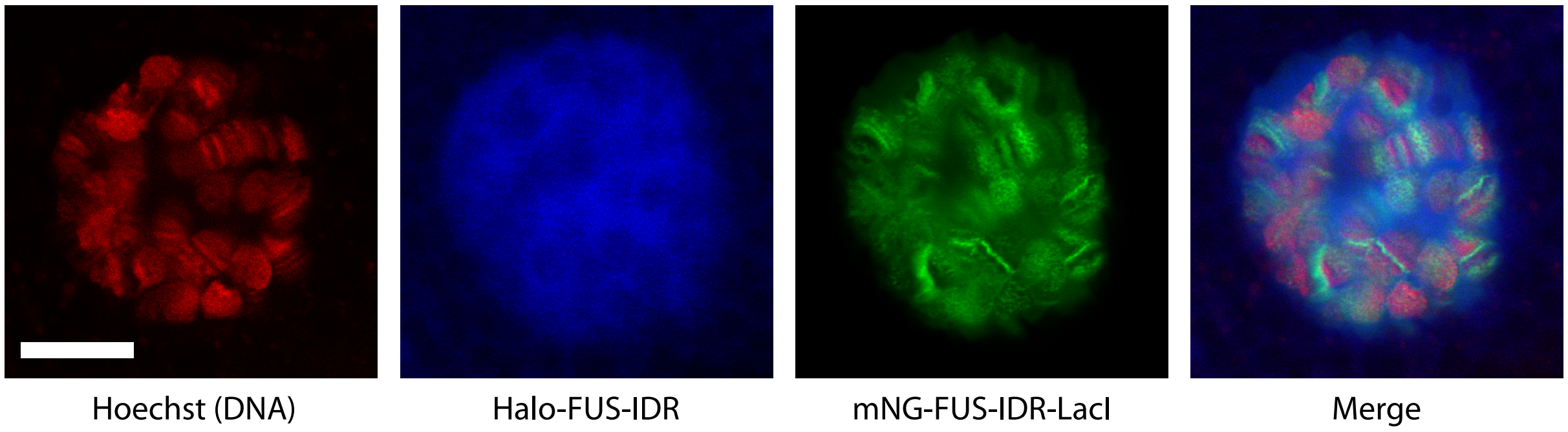

Localization of mNG-FUS-IDR-LacI and Halo-FUS-IDR on polytene chromosomes. Three-color Airyscan image in a salivary gland nucleus expressing UAS-mNG-FUS-IDR-LacI and UAS-Halo-FUS-IDR transgenes driven by an sgs3-GAL4 driver. DNA was counterstained with Hoechst. mNG-FUS-IDR-LacI mostly localizes to euchromatic (DNA-poor) areas and is excluded from heterochromatic (DNA-dense) regions. Scale bar, 10 µm.

Fig. S17.

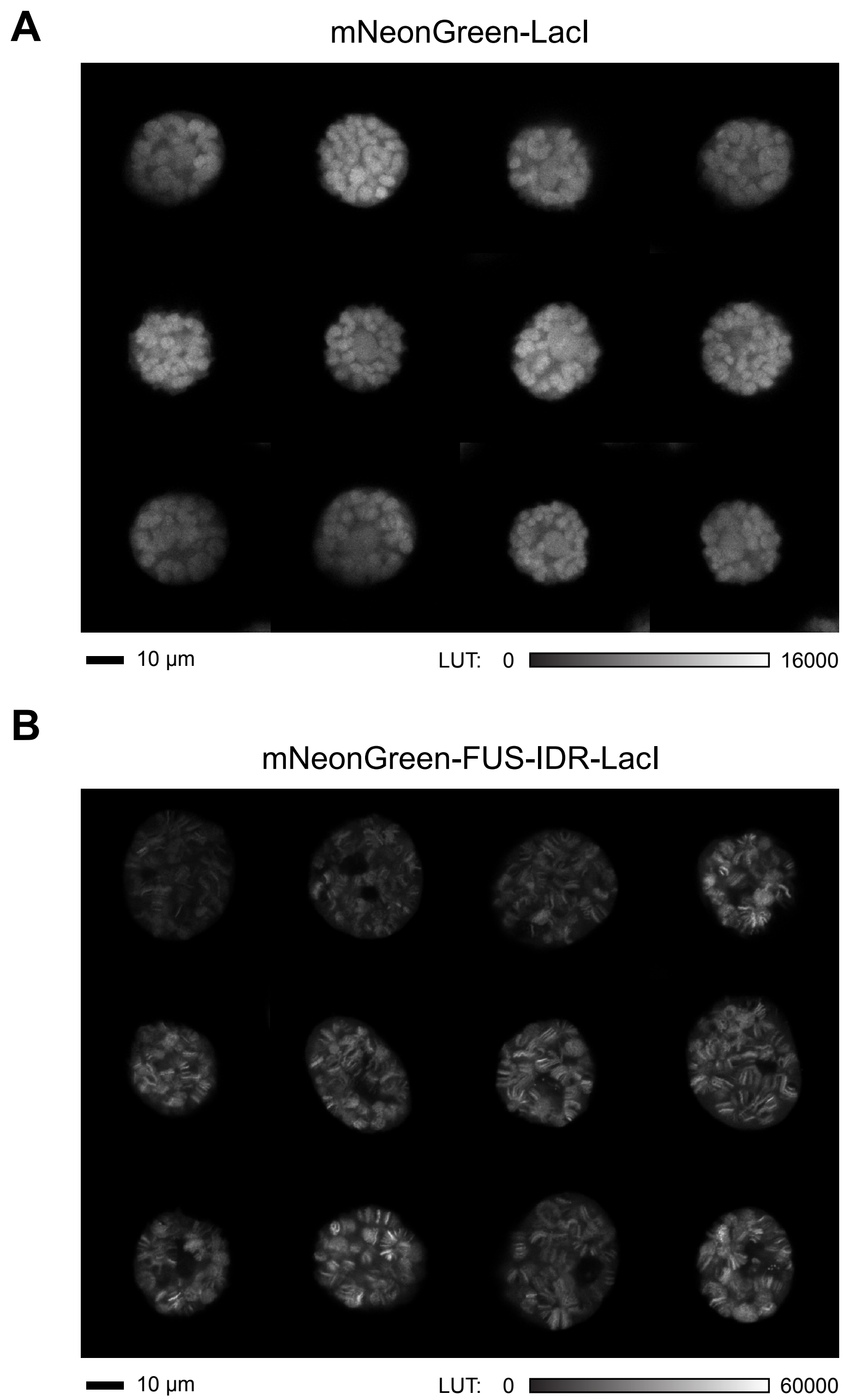

Distribution of mNeonGreen-LacI and mNeonGreen-FUS-IDR-LacI on polytene chromosomes in *Drosophila* salivary gland cells. Both UAS transgenes were driven by the salivary gland-specific sgs3-GAL4 driver line. Imaging settings were identical, and minimum (black) and maximum (white) values in display look-up tables are indicated. The expression level of the mNG-FUS-IDR-LacI transgene (B) was higher under these conditions than that of the mNG-LacI transgene (A).

Fig. S18.

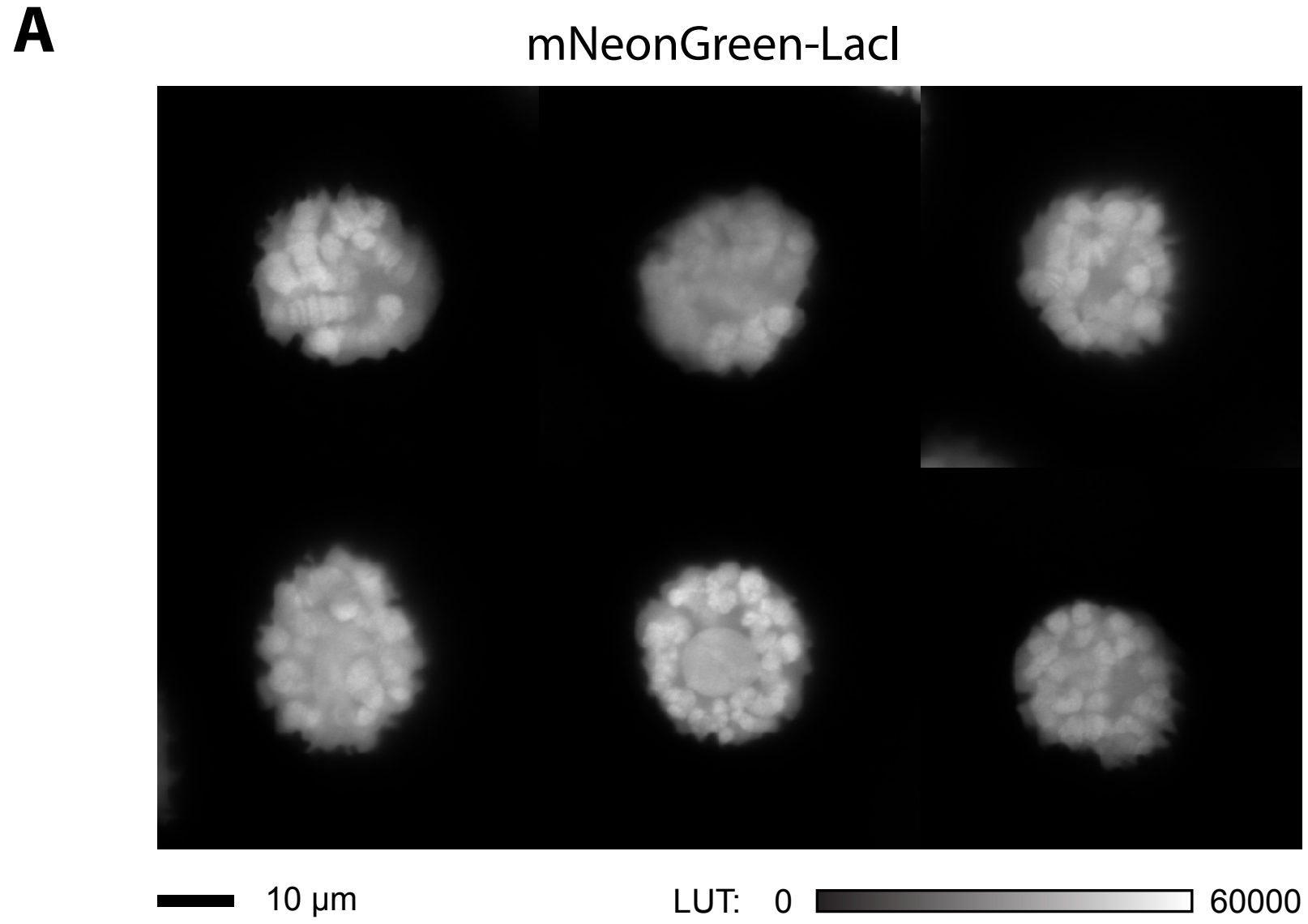

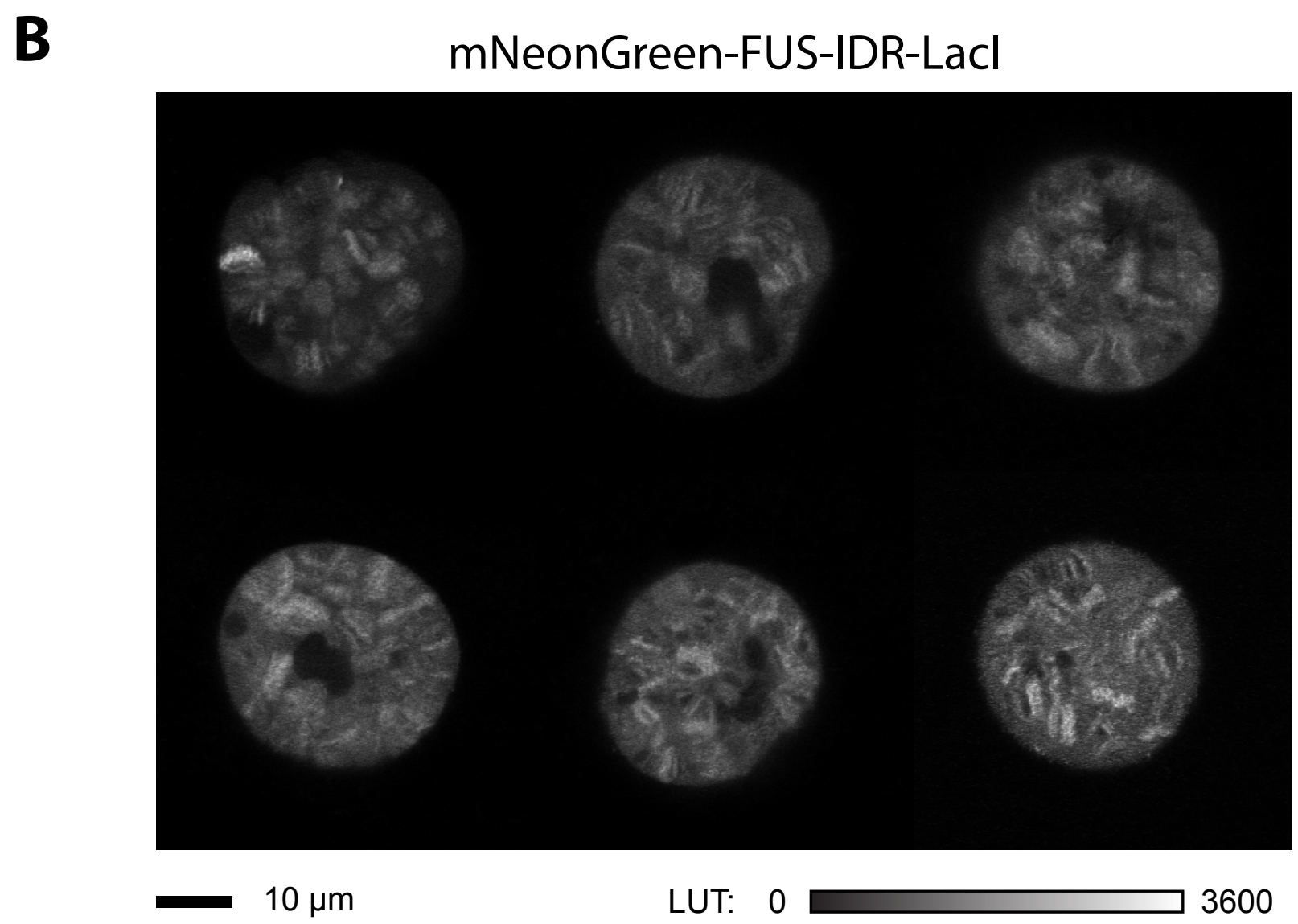

Distribution of mNeonGreen-LacI and mNeonGreen-FUS-IDR-LacI (reduced expression level) on polytene chromosomes in *Drosophila* salivary gland cells. The UAS-mNG-LacI transgene was driven by the salivary gland-specific sgs3-GAL4 driver line, while the UAS-mNG-FUS-IDR-LacI transgene was driven by the GeneSwitch-GAL4 GSG952 driver (BDSC #40984), which yielded weaker expression in salivary gland cells. Imaging settings were identical, and minimum (black) and maximum (white) values in display look-up tables are indicated. The expression level of mNG-FUS-IDR-LacI (B) was substantially lower than that of mNG-LacI (A) under these conditions, yet discrete bands were still apparent.

Fig. S19.

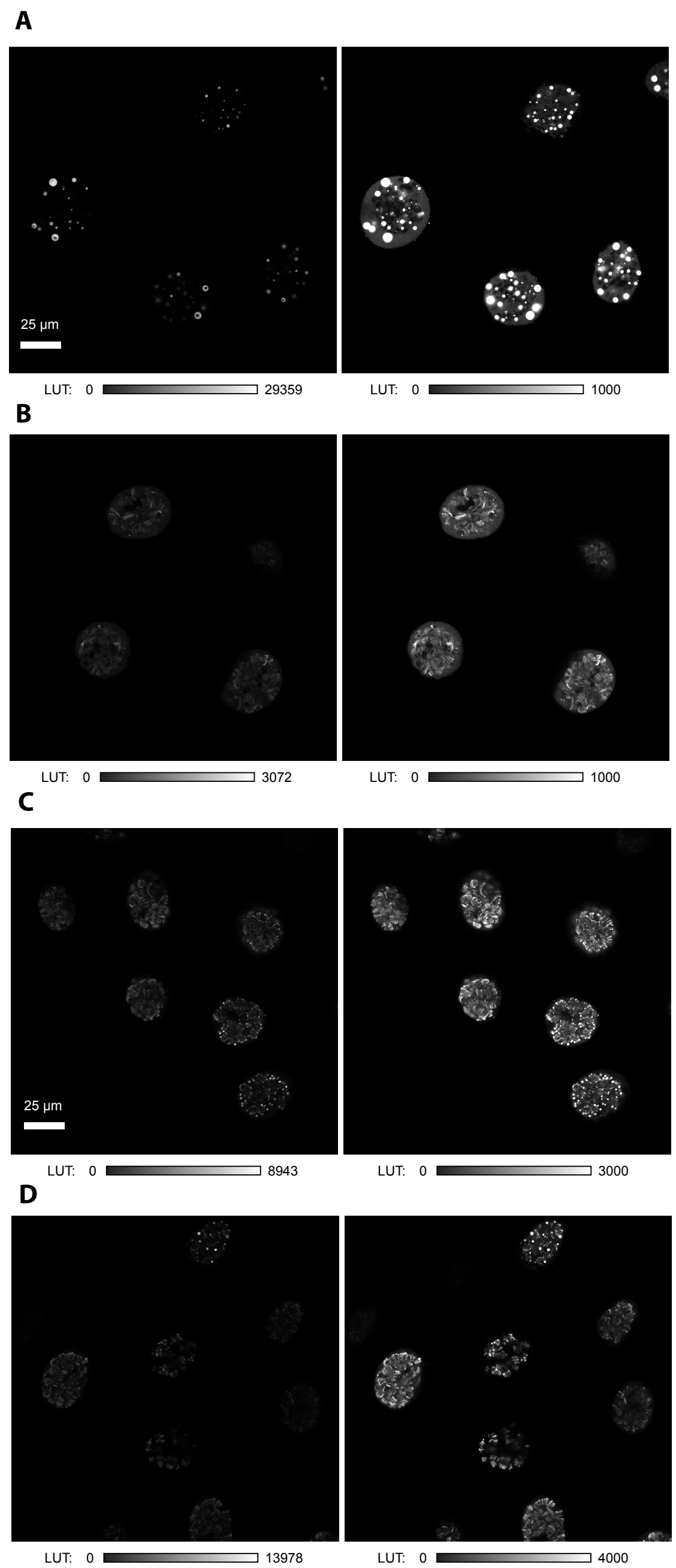

Strong expression of mNeonGreen-FUS-IDR-LacI drives apparent liquid-liquid phase separation. A distinct P element insertion of the UAS-mNG-FUS-IDR-LacI transgene was expressed at high, variable levels when driven by sgs3-GAL4. Large round structures (putative liquid droplets) were observed in the highest-expressing cells (A), while bands were observed in cells with a lower expression level (B). Intermediate cells were also observed (C and D) with a mixture of bands and droplets. Imaging conditions were identical for all images, and lookup tables are displayed for each. In each case, the same image is displayed with a full-range lookup table on the left side and a contrast-enhanced lookup table on the right side.

Fig. S20.

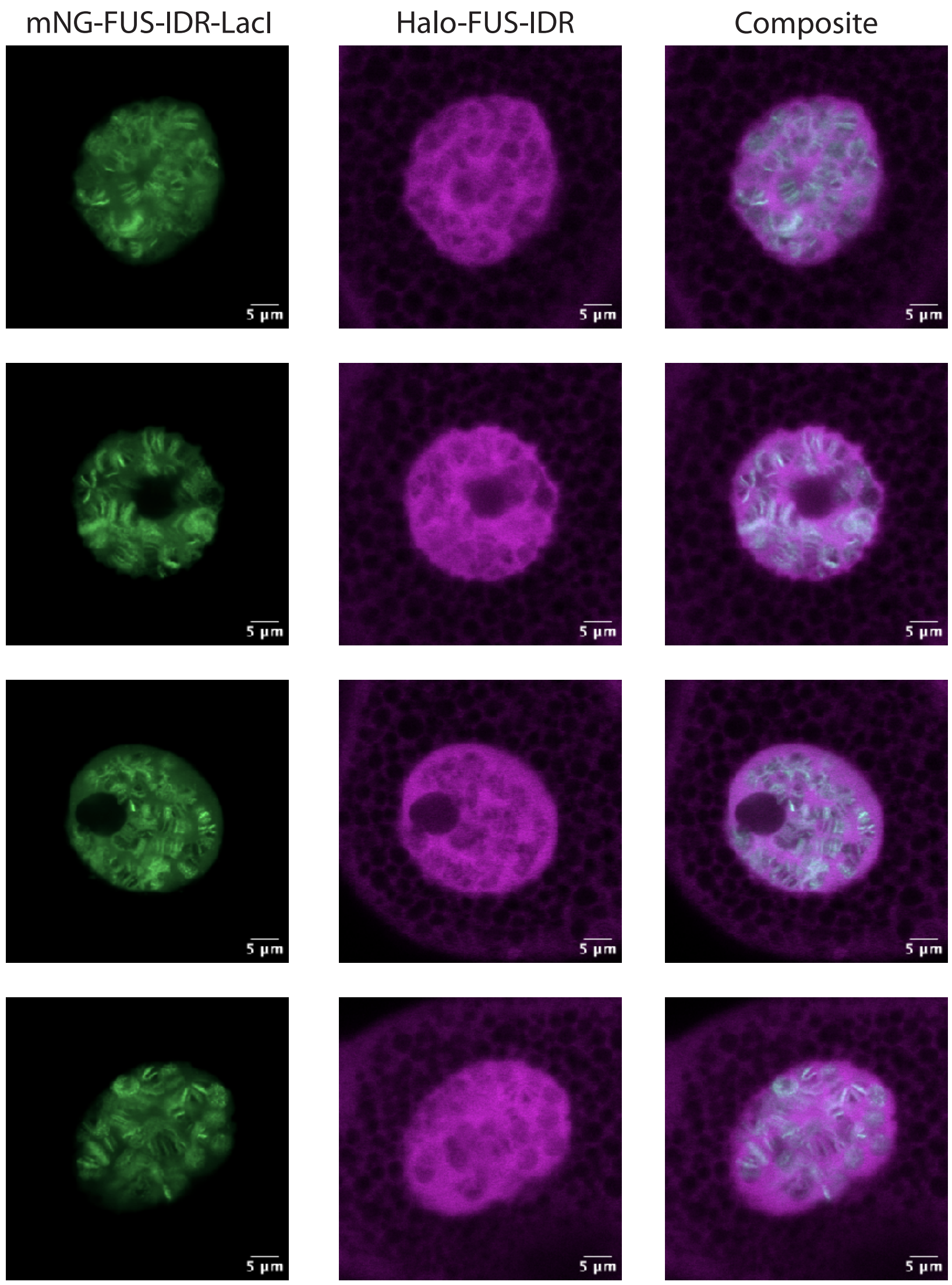

Distinct localization of mNG-FUS-IDR-LacI and Halo-FUS-IDR. Montage of images of salivary gland cells expressing UAS-mNG-FUS-IDR-LacI and UAS-Halo-FUS-IDR transgenes driven by an sgs3-GAL4 driver. Halo-FUS-IDR is mostly nucleoplasmic but shows some weak co-localization with mNG-FUS-IDR-LacI bands on polytene chromosomes.

Fig. S21.

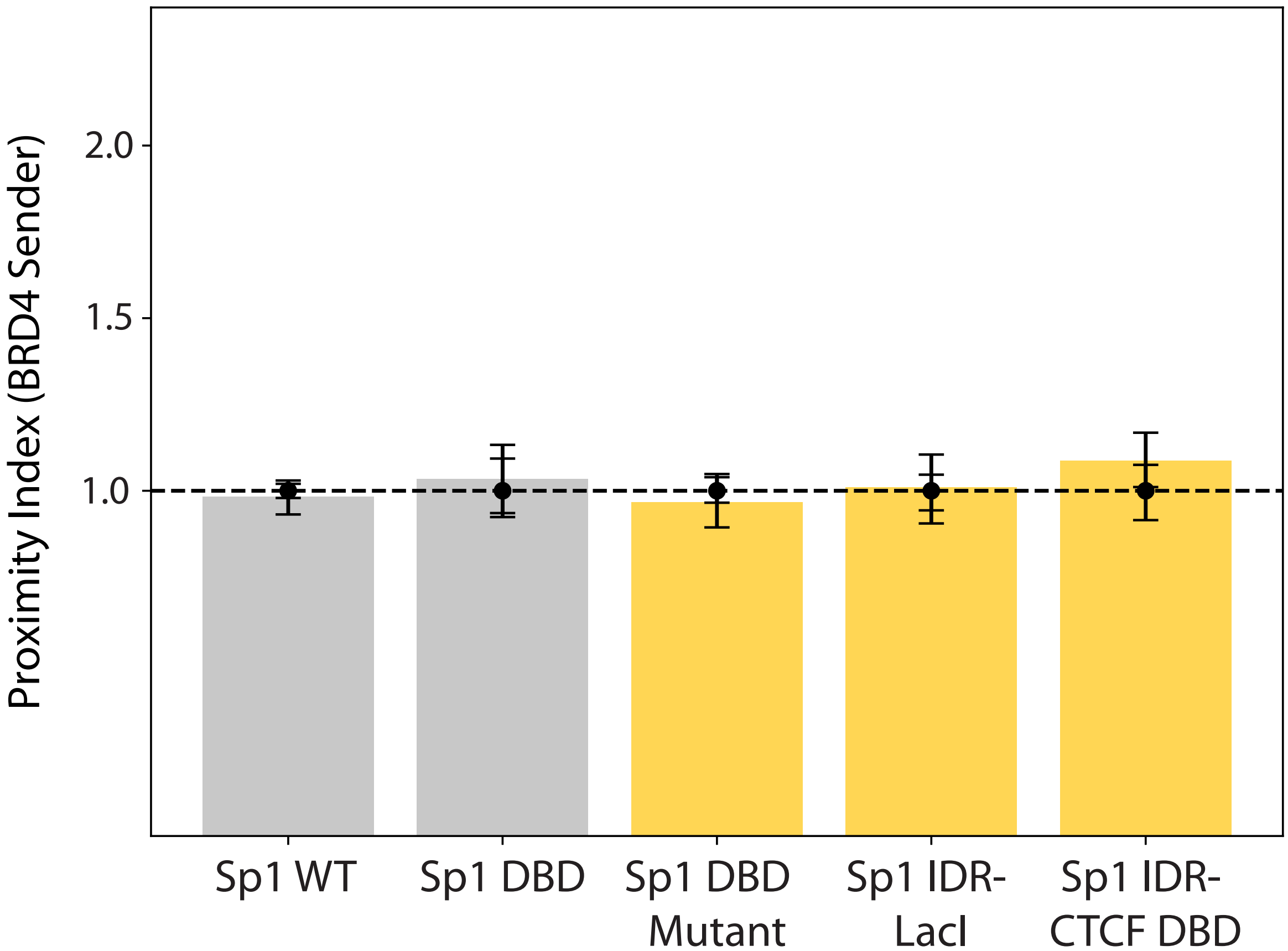

Sp1's IDR does not bind BRD4. PAPA proximity index between endogenous Halo-BRD4 and SNAPf-fusions of Sp1 WT, Sp1 DBD, Sp1 DBD Mutant, Sp1 IDR-LacI, or Sp1 IDR-CTCF DBD. See (*21*) for detection by PAPA of a known BRD4 interaction with P-TEFb.

Table S1. (separate file)

(Sheet A) Sample sizes for SMT-based state-array analysis. Sender and receiver constructs are indicated. "Cells" counts the number of nuclei analyzed, and "Trajectories" counts the number of single-molecule trajectories with two or more detections included in state-array analysis after green reactivation (PAPA) or violet reactivation (DR).
(Sheet B) Sample sizes for PAPA proximity index bar plots shown in the main and supplementary figures. Sender and receiver constructs are indicated. "Cells" counts the number of nuclei analyzed.

Table S2. (separate file)

ChIP-seq and CUT&RUN library details and read numbers.

Table S3. (separate file)

Reproducible peaks identified by CUT&RUN (IDR score ≤ 0.1). Number of CUT&RUN peaks called by MACS3 that passed a reproducibility test (IDR score ≤ 0.1) in two biological replicates for the specified sample and antibody. CTRL: U2OS V5-Halo-Sp1 endogenous knock-in line. The last two columns indicate the percent of peaks overlapping with either FLAG CUT&RUN peaks in the inducible SP1 WT line or CTCF ChIP-seq peaks in the CTRL line.

Table S4. (separate file)

List of DNA constructs and antibodies used in this study.

Data S1. (separate file)

GenBank sequences of the DNA constructs used in this study (see also Table S4).
